# How Does Inattention Influence the Robustness and Efficiency of Adaptive Procedures in the Context of Psychoacoustic Assessments via Smartphone?

**DOI:** 10.1177/23312165241288051

**Published:** 2024-11-18

**Authors:** Chen Xu, David Hülsmeier, Mareike Buhl, Birger Kollmeier

**Affiliations:** 1 Medizinische Physik and Cluster of Excellence Hearing4all, Universität Oldenburg, Oldenburg, Germany

**Keywords:** inattention model, mobile listening test, model-free adaptive procedure, Monte-Carlo simulations

## Abstract

Inattention plays a critical role in the accuracy of threshold measurements, e.g., when using mobile devices. To describe the influence of distraction, long- and short-term inattention models based on either a stationary or a non-stationary psychometric function were developed and used to generate three simulated listeners: fully-, moderately-, and non-concentrated listeners. Six established adaptive procedures were assessed via Monte-Carlo simulations in combination with the inattention models and compared with a newly proposed method: the graded response bracketing procedure (GRaBr). Robustness was examined by bias and root mean square error between the “true” and estimated thresholds while efficiency was evaluated using rates of convergence and a normalized efficiency index. The findings show that inattention has a detrimental impact on adaptive procedure performance—especially for the short-term inattentive listener—and that several model-based procedures relying on a consistent response behavior of the listener are prone to errors owing to inattention. The model-free procedure GRaBr, on the other hand, is considerably robust and efficient in spite of the (assumed) inattention. As a result, adaptive techniques with desired properties (i.e., high robustness and efficiency) as revealed in our simulations—such as GRaBr—appear to be advantageous for mobile devices or in laboratory tests with untrained subjects.

## Introduction

Measuring sensory thresholds is one of the fundamental topics in psychophysics and central for hearing assessment, e.g., in hearing screening, in characterizing auditory functions, or in rehabilitative audiology. There are many methods established to obtain threshold measurements efficiently, including various adaptive procedures that steer the stimulus level according to the previous responses of the participant (Leek, 2001; Treutwein, 1995). Psychophysical procedures that are used to calculate sensory thresholds typically rely on participants to be attentive so that they can produce consistent responses. However, Green (1995), observed that participants can be inattentive and produce responses that are unrelated to the stimulus (Wichmann & Hill, 2001), and modeled sustained inattention by adjusting the lapse rate of the psychometric function. Green's (1955) stationary inattention model (herein referred to as “long-term inattention”) has been widely adopted by many other studies to evaluate the robustness of the adaptive procedures against inattention (e.g., Manning et al., 2018; Rinderknecht et al., 2018; Shen & Richards, 2012). However, with the recent advent of remote, self-driven, and even smartphone-based hearing testing, a completely different setting of threshold measurements comes into play (e.g., Bisitz & Silzle, 2011; Luengen et al., 2021; Ooster et al., 2020). Such occasional inattention—termed “short-term inattention”—requires a different, non-stationary attention model, where the individual state of attention is randomly drawn to subsequently determine the respective response probability. This differs from sustained inattention, which is modeled using a fixed, stationary probability.

The study aims to unravel how this type of assumed short-term inattention influences the result of the various hearing threshold measurement procedures in contrast to long-term inattention behavior known from the literature (Green, 1995; Rinderknecht et al., 2018). The second aim is to quantify, normalize, and eventually optimize the robustness and efficiency of the adaptive procedures to be used for smartphone measurements in the future. This is a prerequisite for our research question: Do adaptive procedures differ in their robustness against both types of inattention and how do these differences affect their efficiency?

To measure auditory thresholds efficiently, Kaernbach (1990) proposed the single interval adjustment matrix (SIAM) approach based on a simple yes-no task for testing. SIAM was validated by Shepherd et al. (2011) using auditory stimuli for its ability to measure absolute threshold quickly, reliably, and accurately for human participants. The SIAM procedure utilizes the outcome of the signal detection matrix (i.e., hit, miss, false alarm, and correct rejection) to adjust the sound level in an adaptive manner. Green (1990, 1993, 1995) and Gu and Green (1994) introduced a single interval adaptive approach employing the maximum likelihood procedure (MLP). The MLP procedure consists of two steps: maximum likelihood estimation and stimulus selection. In the maximum likelihood estimation, different psychometric functions are proposed as hypotheses. Then the likelihood of each hypothesis is calculated and the function with the highest likelihood is selected to obtain the level of the next trial from the inverse function at the p-target, i.e., the threshold level that corresponds to the (target) probability p at the estimated psychometric function (e.g., 50% for a yes–no task, and 75% for the two-alternative forced choice (2AFC) task, see Grassi & Soranzo, 2009, respectively, as typical examples from a certain range of values). The MLP method appears to be rather efficient and was validated with human subjects by Amitay et al. (2006) and Leek et al. (2000). However, Green (1995) found that the MLP yielded a poor estimate of thresholds if participants were inattentive. This “unforgiving” property of model-based or parametric methods results from the fact that the whole track history influences the respective next-level placement. This motivated the introduction of hybrid methods (e.g., Hall, 1981) where an adaptive, non-parametric level placement procedure with a shorter memory is combined with a maximum likelihood (ML) method for the final threshold estimate. More recently, Shen and Richards (2012) optimized the original MLP method and designed an updated maximum likelihood (UML) procedure, aiming at improving the low accuracy of threshold estimates resulting from lapses in attention. In the UML procedure, the stimulus selection process takes the interim estimate of the lapse rate into account.

The adaptive method parameter estimation by sequential testing (PEST) is among the first non-parametric adaptive psychophysical testing methods (Gescheider, 2013; Taylor & Creelman, 1967). The PEST method compares the respective correct response rate with the target probability and determines the level of the subsequent stimulus by interpolation using a series of diminishing step sizes. The same long-term memory problem as with the MLP methods exists for Bayesian adaptive procedures that build upon the PEST method such as best PEST (Pentland, 1980), QUEST (Watson & Pelli, 1983), and the state-of-the-art QUEST+ approach (Watson, 2017). QUEST+ utilizes the minimum entropy principle to select the respective next stimulus level and maximum likelihood theory to estimate the final values of the parameters. Specifically, QUEST+ searches for the most informative stimulus by minimizing the entropy of the posterior probability density. When taking the interim estimate of lapse rate during the stimulus selection process into consideration, these methods including UML are not as “unforgiving” as those discussed before and, hence, achieve accurate and efficient threshold estimation inside the lab (Watson, 2017).

One problem of the single-interval Yes/No procedure (e.g., MLP in Gu & Green, 1994)—when used in combination with the adaptive rules discussed so far—is the need to control (or at least to detect) the individual detection criterion as described by signal detection theory (Green & Swets, 1966). This is usually done by inserting sham trials (also referred to as “catch trials” in psychology), i.e., trials that do not contain a signal to estimate the false alarm rate concurrently with the correct detection rate. Alternatively, n-interval forced-choice (nIFC) methods are used where only one randomly selected interval contains the target signal and the other (n-1) intervals the reference. However, the necessity of these additional blank intervals (as well as sham trials) increases the measurement time and thus reduces the efficiency of the procedure for estimating thresholds. Furthermore, naïve subjects might get frustrated if they do not perceive the intended signal frequently, as pointed out by Lecluyse and Meddis (2009), and tend to lose the cue for stable detection. This calls for a minimum of sham trials or blank intervals and for providing suprathreshold stimulus levels not too rarely during a track.

To accommodate both requests, Lecluyse and Meddis (2009) and Meddis and Lecluyse (2011) recommend the single interval up and down (SIUD) procedure for tone detection. The SIUD involves presenting two tones—the probe tone and an additional cue tone with a fixed level increase of 10 dB—while participants indicate how many tones they have heard (0, 1, or 2 tones). The responses are used to track the threshold of the probe tone and to detect false alarms recorded in (rare) sham trials where the cue tone is absent, leading to an abortion of the track. Although human experiments suggest that the SIUD procedure is accurate and efficient for threshold measurement, there is no systematic assessment of the influence of inattention on the robustness and efficiency of this procedure. Also, the presentation of the cue tone requires a significant amount of measurement time. Additionally, as the level of the cue tone exceeds the probe tone level by a fixed amount of 10 dB, this difference might be appropriate for the initial portion of the adaptive track, but too large for the final portion to be helpful and informative for the determination of the threshold as the cue tones are always audible in the final portion of the adaptive track.

In the SIUD procedure, the information about the audibility of the cue tone with the (much) higher level is discarded in regular, non-sham trials. This has no negative effect on the outcome of the threshold estimation process since the cue tone audibility information relates to the saturation region of the psychometric function at approximately 100% which does not decrease the uncertainty about the threshold level. However, discarding the information to be gained from the cue tones in most trials (i.e., nearly 50% of the stimuli presented) could result in a poorer efficiency of the procedure in terms of a decrease in measurement uncertainty per unit of measurement time spent.

We therefore suggest a smaller, adaptively adjusted difference between the probe and cue tone in order to “bracket” the threshold and to exploit the detectability of the cue tone by the tracking procedure as well. This is expected to increase the efficiency of the procedure as more cue tones are presented near the threshold level. Hence, based on the SIUD procedure proposed by Lecluyse and Meddis (2009), we suggest the Graded Response Bracketing procedure (GRaBr), and compare the GRaBr procedure with the procedures discussed so far.

Although adaptive, response-criterion-compensating procedures (i.e., SIAM, MLP, UML, QUEST+, SIUD, and GRaBr) are advantageous in laboratory measurements and efficient for achieving a certain level of accuracy, sometimes they are not assigned importance in clinical practice for pure-tone threshold estimation (Lecluyse & Meddis, 2009). Instead, practitioners in audiology or otolaryngology make a compromise between speed and simplicity versus accuracy. They primarily use manual methods for pure-tone threshold estimation such as the Hughson–Westlake procedure because of its simple administration, little patient training, and easy implementation (Hughson et al., 1944). Bisitz and Silzle (2011) reported a self-administered threshold measurement approach, i.e., the APTA procedure, which is based on the Hughson-Westlake method, whose modified versions are widely applied in clinical audiogram measurements (Guo et al., 2021; Hughson et al., 1944). It is an ascending method (i.e., the procedure typically starts with an inaudible sound level and gradually increases the sound level until the participants indicate they can hear the tone) to assess the listener's hearing threshold, where the listener’s task is to indicate whether a tone is heard or not.

Taken together, a number of well-motivated, established procedures for adaptively testing thresholds exist for laboratory use, but a consistent comparison with respect to their efficiency and robustness against long- and short-term inattention is still missing. This, however, is an important prerequisite for selecting the most appropriate procedure for mobile testing. To address this gap, we performed Monte Carlo simulations for the adaptive procedures listed above, i.e., APTA, GRaBr, MLP, QUEST+, SIAM, SIUD, and UML, while systematically varying the two modes of inattention. The performance of all adaptive procedures was evaluated in terms of the robustness of the (simulated) observable against inattention, as well as their normalized efficiency which accounts for the “effective” time used to derive a threshold estimate including any sham trials or aborted tracks.

## Methods

### Inattention Model

The behavior of a virtual listener is typically modeled with a stationary psychometric function, i.e., the relationship between stimulus intensity (e.g., sound level) and a test subject's response (e.g., the proportion of “yes” responses). The psychometric function was formulated as a four-parameter transformed logistic function by Brand and Kollmeier (2002) and Green and Swets (1966):

(1) $$p(L,\varphi )=p_{\min}+(p_{\max}-p_{\min})/(1+e^{-4s(L-L_{50})})$$

where p is the probability of “yes” responses, L defines the sound level, and φ describes the parameter vector. p_min_ indicates the lower boundary of the function (also referred to as the false alarm rate for a yes/no paradigm). p_max_ denotes the upper asymptote of the function (the miss rate is calculated by 1–p_max_). “s” is the slope of the function at the half-way point. L_50_ describes the threshold at the half-way point between the minimum and maximum of the psychometric function. The default parameter vector φ was set as (p_min _= 0, p_max _= 1, s = 0.125, L_50 _= 15), where the target threshold was 15 dB.

**Figure 1.**
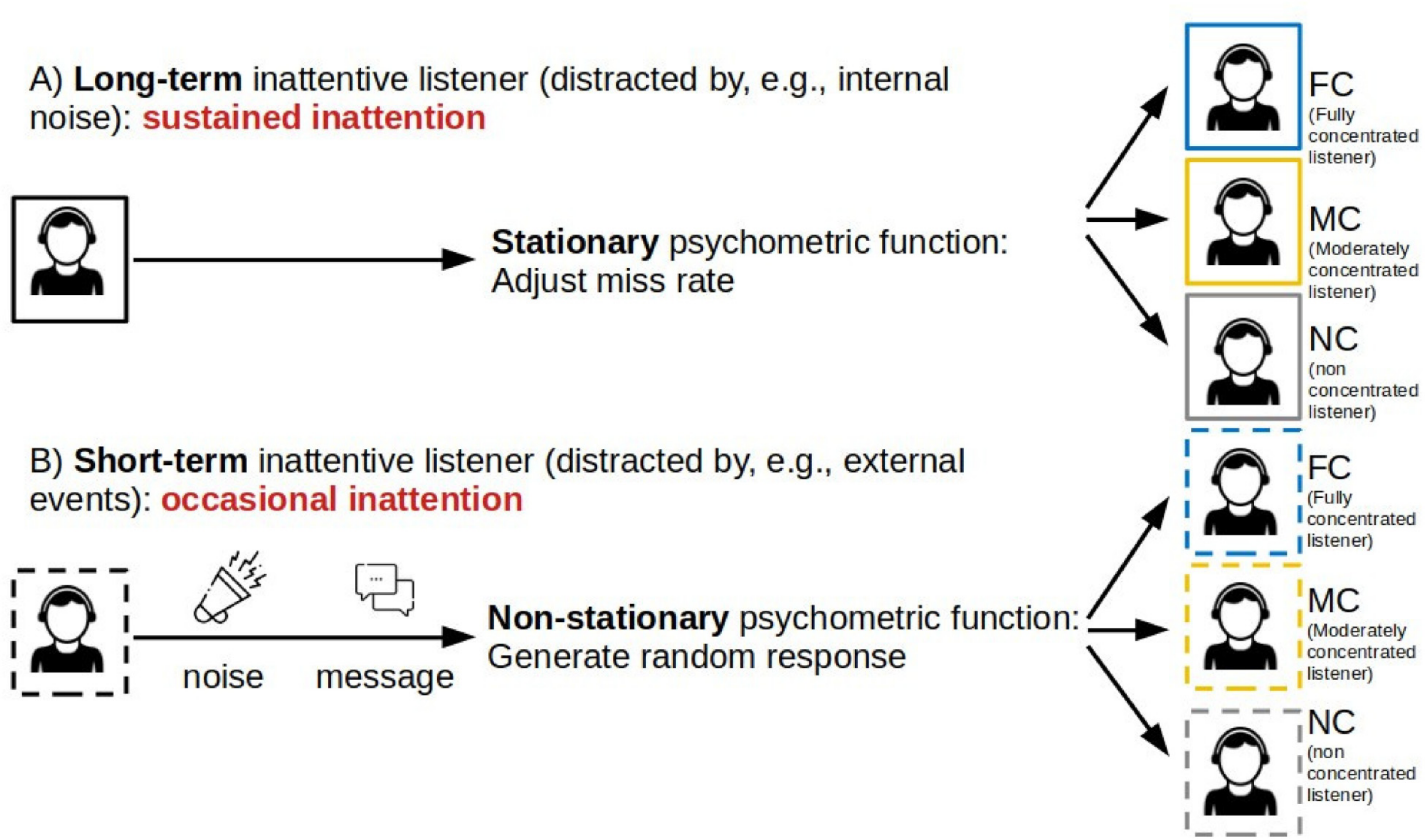
Inattentive listener simulation. (A) long-term inattentive listener (solid boxes), (B) short-term inattentive listener (dashed boxes). FC: Fully concentrated listener, MC: Moderately concentrated listener, NC: Non-concentrated listener. The parameter p_max_ of the long-term FC, MC, and NC listeners is set to 1, 0.95, and 0.9 respectively. The short-term FC, MC, and NC listeners respond randomly in 0%, 10%, and 20% of trials (corresponding to the parameter p_inatt_), respectively.

As illustrated in Figure 1A, we define the inattentive listener distracted by internal noise as a “long-term inattentive listener” exhibiting sustained inattention. This characterization assumes a stationary probabilistic process that persists throughout the entire measurement track. Conversely, in Figure 1B, we describe the inattentive listener as a “short-term inattentive listener” with occasional lapses in attention, where we assume a non-stationary behavior characterized by sporadic bursts of inattention during the measurement track. Note that—averaged across a whole measurement track—the average effect of the short-term inattention model would be reflected in a deformation of the average psychometric function (i.e., an increase of the lower asymptote and decrease of the upper asymptote for a yes-no task) which resembles the psychometric function already employed by the long-term inattentive listener model. However, the interaction between the adaptive procedure and a non-stationary psychometric function (modeled here as a nested random process) would not adequately be covered by the long-term inattentive listener model which uses the same psychometric function in all (simulated) trials. Moreover, the long-term inattention model assumes that the likelihood of a “yes” response under inattention p(“yes”|inattention) = p_min_ whereas the short-term inattention model allows p(“yes”|inattention) to be specified as an arbitrary probability. For participants using smartphones to perform listening tests, it is likely that inattentiveness causes them to respond with “yes” (p(“yes”|inattention) = 1) or “no” (p(“yes”|inattention) = 0) with equal probability. Therefore, in this scenario, p(“yes”|inattention) may differ from the value of p_min_, provided by the long-term inattention model, and instead be closer to 0.5.

**Figure 2.**
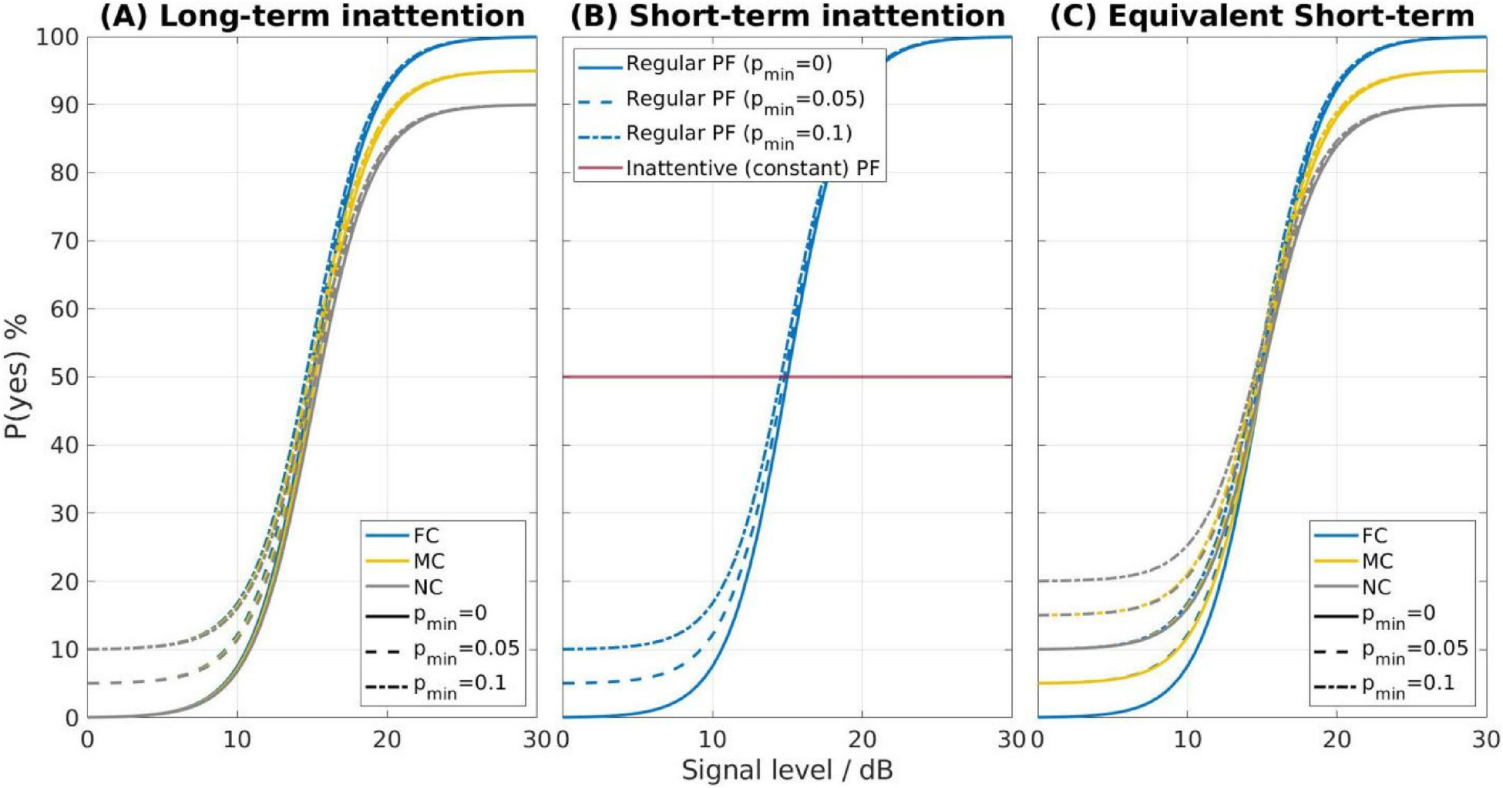
Observer model for (A) long-term inattention, (B) short-term inattention, and (C) equivalent short-term inattention. In the long-term inattention model, p_max_ is set to 1, 0.95, and 0.9 for the FC, MC, and NC listeners. In the short-term inattention model, a constant psychometric function (PF), i.e., p(“yes”|inattention) = 0.5 is employed for up to 20% of all trials (p_inatt _= 0, 0.1 or 0.2), denoted as the FC, MC, and NC listener, respectively, while a regular logistic PF is applied in the remaining trials. This results in the “equivalent expected PF” for short-term inattention in (C), i.e., the expectation value of the PF from the nested random process of the short-term inattention model. In addition, three levels of false alarm rate p_min_ (0, 0.05, and 0.1) are included for both long- and short-term inattention models.

The psychometric functions (PF) generated for both types of inattentive listeners are illustrated in Figure 2(A) for the long-term and (B) for the short-term inattentive listener. The variations in the rate of inattention for both types of inattentive listeners are termed non-, moderately-, and fully-concentrated listeners (abbreviated as NC, MC, and FC listeners, respectively). The FC listener as a reference group is identical for both types of inattentive listeners. In the case of long-term inattention, following Green (1995), we vary the inattention by setting the upper asymptote p_max_ of the regular PF from 0.9 to 1.0 with a spacing of 0.05 (i.e., 0.9, 0.95, and 1.0). For short-term inattention, a regular PF is assumed in most trials, while in up to 20% of all trials (i.e., p_inatt _= 0, 0.1 or 0.2), a random response behavior is foreseen, i.e., a constant PF with p(“yes”|inattention) = 0.5. This value of p(“yes”|inattention) = 0.5 is chosen as the most likely value if no information about the listener is available. It also illustrates the potential impact of scenarios where p(“yes”|inattention) is higher than any of the p_min_ values implemented in the current study.

For both types of inattention, we also vary the lower asymptote p_min_ between 0 and 0.1 with a 0.05 step size (i.e., 0, 0.05, and 0.1) to account for different false alarm rates (in case of a yes/no paradigm), as shown in Figure 2. A total of 18 simulated listeners are established: Three levels of inattention (FC, MC, and NC listeners) × Three levels of false alarm rate (0, 0.05, and 0.1) × Two types of inattention (long-term and short-term). Note that a symmetric shape of the PF (i.e., p_max _= 1–p_min_) is assumed in six of these simulated listeners whereas the more general case of an asymmetric PF is assumed for 12 of them.

The short-term inattention observer model switches randomly between a regular and a constant psychometric function. Mathematically, a nested process with two states using a conditional function is employed. In the i_th_ trial, a random decision is first made to determine the state (i.e., a state representing decision-making based on the sensory input if i belongs to the set Q with P(i ∈ Q) = 1-p_inatt_ and a state for randomly guessing if i belongs to the complementary set $\bar{Q}$ with P(i ∈ $\bar{Q}\bar{Q}$ ) p_inatt_). Subsequently, a random decision is made if the respective response is “yes” or “no,” which is controlled by the respective conditional psychometric function given as:

(2) $$p(L,\varphi ,i{)}_{\mathrm{short}}\;=\;\{\begin{array}{c} \;p_{\min}+\frac{1-p_{\min}}{1+e^{-4s(L-L_{50})}}\;if\;i\;\in \;Q\\ 0.5\;if\;i\;\in \;\bar{Q} \end{array}$$

The long-term equivalent of the PF from (2)—loosely denoted as “average” PF across a whole track—is given as the expectation value from this nested random process. We denote this expectation value of the PF effectively resulting across a whole track as the “equivalent expected PF” for short-term inattention, which is given in Figure 2(C). Note that it does not approach unity for the upper asymptote, but rather the value 1–p_inatt_/2. Likewise, the lower asymptote does not approach p_min_, but rather is increased by the value p_inatt_/2:

$$p_{max,short}\;=1\;-\;p_{inatt}/2$$

(3) $$p_{min,short}\;=p_{min}+p_{inatt}/2$$

Hence, the long-term behavior of the PF for the short-term inattentive listener—expressed by the equivalent expected PF—is very similar to the psychometric function employed for the long-term inattention model (if p_max,short_ is replaced by p_max_ and if p_min,short_ is replaced by p_min_). However, the trial-by-trial behavior differs considerably between the long- and short-term inattention models. The single-trial PF shape of the long-term inattention model is trial-independent as it does not vary throughout a track whereas the PF shape of the short-term inattention model varies from trial to trial as the listener randomly switches between two different states.

When comparing Figure 2(A) and (C), the upper asymptote p_max,short_ of the equivalent expected PF for short-term inattention is in line with p_max_ of the long-term inattentive listener due to the respective choice of the parameter p_inatt_ for the FC, MC and NC listener. However, there is a discrepancy in the lower asymptote between p_min_ and p_min,short_. Therefore, the equivalent expected PF of the short-term inattention model cannot be made equal to the long-term inattention model at the same level of inattention and false alarm rate. An equivalence can only be made if the short-term inattention model is compared to the long-term inattention model at the same level of inattention but at different levels of false alarm rate, e.g., the short-term MC listener with p_min _= 0 should be compared with the long-term MC listener with p_min _= 0.05.

Additionally, when the constant PF in the short-term inattention model equals the false alarm rate, the short-term inattention model would be equivalent to the long-term inattention model. This assumes that participants’ decision-making during inattentive trials still follows the same sensory process as in attentive trials, i.e., all trials could be described by the well-established pure sensory process outlined in Green (1995). However, in this study, we assume during inattention a uniform distribution of responses with a constant PF (i.e., p(“yes”|inattention) = 0.5) which does not necessarily equal the false alarm rate. Note that this uniform distribution in inattentive trials follows from the maximum uncertainty or minimum entropy principle and assumes that participants generally make judgments independently of stimulus intensity in the inattentive trials. Thus, the whole process is rather described as a nested model of two independent processes. Please see the Appendix for the methods and results of the hybrid inattention model (i.e., a listener is distracted both by long- and short-term inattention simultaneously).

### Adaptive Procedures

**Figure 3.**
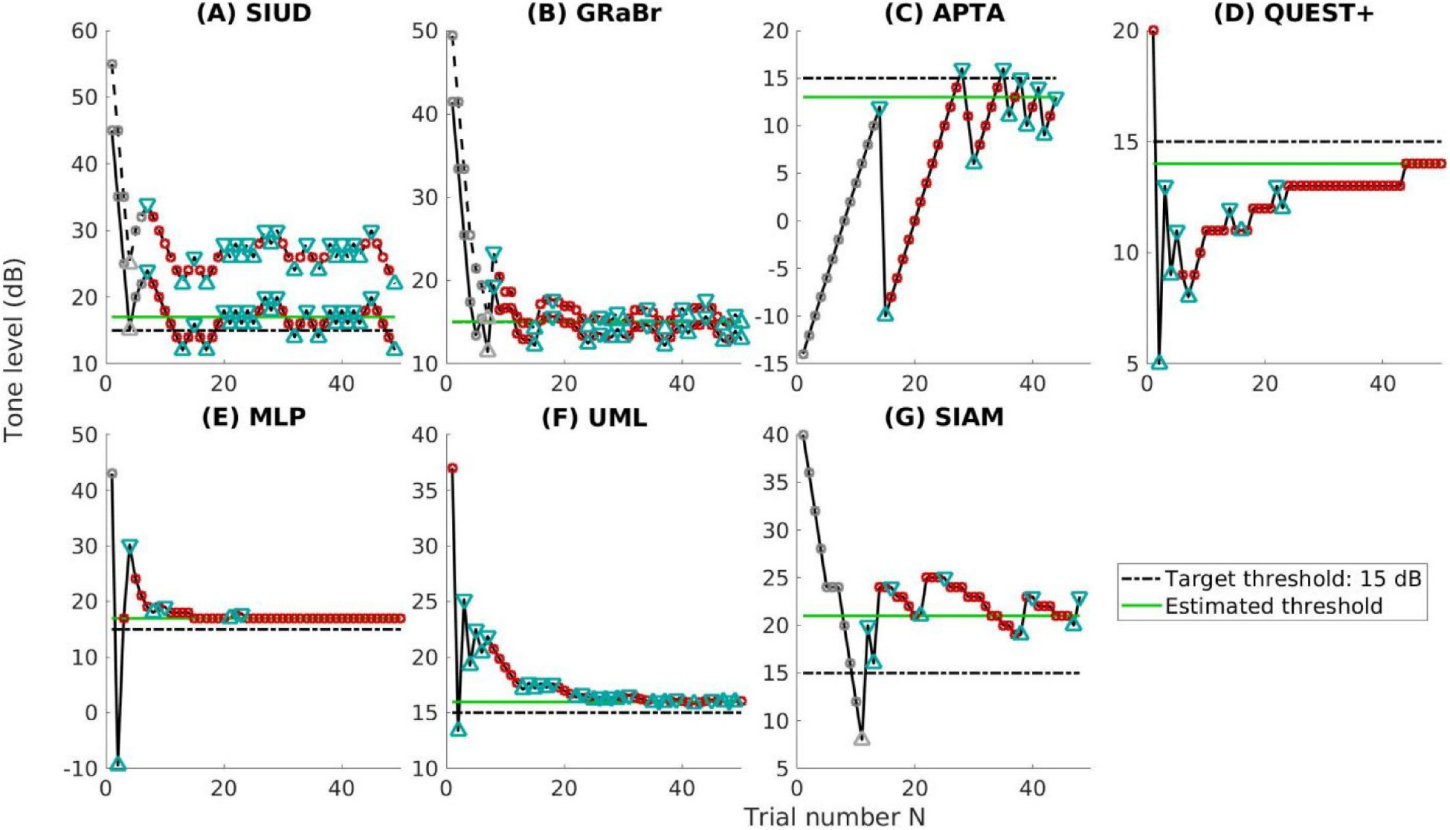
Examples for an adaptive run, i.e., tone level as a function of the number of trials for the seven procedures considered here. (A) SIUD = single interval up and down (Lecluyse & Meddis, 2009), (B) GRaBr = graded response bracketing, (C) APTA = automated pure tone audiometry (Bisitz & Silzle, 2011), (D) QUEST +  (Watson, 2017), (E) MLP = maximum likelihood procedure (Green, 1993), (F) UML = updated maximum likelihood (Shen & Richards, 2012), and (G) SIAM = Single Interval Adjustment Matrix (Kaernbach, 1990). Green line: threshold estimate. Triangles: trials at a reversal point. Grey circles: discarded trials for threshold estimation. Red circle: active trials. Solid line: main track for threshold estimation. Dashed line: level track of the cue tone trials. Dot-dashed line: target threshold 15 dB.

The properties of seven employed adaptive procedures that will be compared in this paper are provided in Table 1 and exemplary tracks are visualized in Figure 3. The target threshold was fixed at 15 dB. MLP, QUEST+, SIAM, and UML are model-based or parametric procedures whereas the other procedures are model-free (Audiffren & Bresciani, 2022). Typically, the parameter space (i.e., ranges of parameters L_50_, s, p_min_, p_max_, as well as procedure-specific parameters) together with the stimulus space (i.e., sound level) are required to be specified beforehand for those model-based procedures. MLP, QUEST+, and UML mainly employ Bayes’ rule for stimulus placement and will herein be referred to as Bayesian procedures. Only APTA is a variant of the clinical method. Most adaptive procedures (e.g., MLP, QUEST+, SIAM, UML, and APTA) utilize a yes/no task, whereas the SIUD and GRaBr utilize a variant of the standard yes/no task (i.e., counting how many tones are detected, with the three response options: none, one, and two tones). Twenty percent catch trials are implemented in SIUD and GRaBr while the other adaptive procedures contain no catch trials. Two intervals are presented in SIUD and GRaBr whereas the other adaptive procedures have only one interval.

**Table 1.** Summarized Characteristics of the Employed Adaptive Procedures.

Procedure	Model based^a^	Bayesian	Clinical	Proportion of catch trails	Number of intervals on each trial	Parameter space	Stimulus space	Literature
SIUD	−	−	−	20%	2	N/A	N/A	Lecluyse and Meddis (2009)
GRaBr	−	−	−	20%	2	N/A	N/A	Present
APTA	−	−	×	−	1	N/A	N/A	Bisitz and Silzle (2011)
QUEST+	×	×	−	−	1	L_50 _= [−10 50] s = 0.125 p_min _= 0 p_max _= 1	[−10, 50]	Watson (2017)
MLP	×	×	−	−	1	L_50 _= [−10 50] s = 0.125 p_min _= [0 0.1] p_max _= 1	[−10, 50]	Green (1993)
UML	×	×	−	−	1	L_50 _= [−10 50] s = 0.125 p_min _= 0 p_max _= 1	[−10, 50]	Shen and Richards (2012)
SIAM	×	−	−	−	1	t = 0.75	N/A	Kaernbach (1990)

^a^ The model-based (i.e., parametric) procedures hypothesize the parameters of the psychometric function while the model-free do not.

The six established adaptive procedures (i.e., SIUD, APTA, QUEST+, MLP, UML, and SIAM) followed as closely as possible the respective protocols introduced by Lecluyse and Meddis (2009), Bisitz and Silzle (2011), Watson (2017), Green (1993), Shen and Richards (2012), and Kaernbach (1990). The starting level was set using the strategy described in Lecluyse and Meddis (2009) for all the procedures to ensure a fair comparison. The starting level of all methods except for APTA and QUEST+ followed a discrete uniform distribution ranging between 35 dB and 45 dB with a step size of 1 dB (11 values). The APTA procedure began at −10 dB SPL (inaudible level) while QUEST+ determined the starting level based on its own rule by averaging over the upper and lower limit of the stimulus range. Thresholds were estimated for each procedure based on the median of all sound levels (indicated with a green line in Figure 3) but the trials before the first reversal were discarded for the SIAM, SIUD, and GRaBr procedures. Please note that APTA followed a specific rule to estimate the threshold (i.e., the sound level of the last “yes” response was determined as the threshold). All procedures were run until the *N* = 50th trial.

#### SIUD

Lecluyse and Meddis (2009) and Meddis and Lecluyse (2011) introduced an adaptive absolute threshold measurement based on a simple variant of the yes/no task: normally two tones were presented in a trial and the listeners were required to count how many tones they heard (zero, one, or two, respectively). One of the tones (denoted as cue tone) had a fixed 10 dB higher level than the probe tone and had a 20% chance to be muted. Catch trials for adaptive procedures were first introduced by Gu and Green (1994) to observe the false alarm rate p_min_ of the psychometric function (i.e., proportion of yes responses to catch trials). The false alarms are used to calculate the “catch out rate” which is defined as the number of false alarms divided by the total number of catch trials. Since Leek et al. (2000) validated that the catch-out rates were small (around 5%), Lecluyse and Meddis (2009) suggested that instead of observing the catch-out rate, the threshold task should restart when a false alarm occurs (this is referred to as an “abortion incident”). Thus, following Lecluyse and Meddis (2009), one track restarted in our study when a false alarm happened. The cue tone levels are visualized with the dashed line in Figure 3A. The step size was set up to 10 dB at the beginning. The sound level was set to the middle point of the previous two levels after the first “one” tone response was recorded. Afterwards, a 2 dB step size was used.

#### GRaBr

The level interval between the two tones of SIUD was always fixed at 10 dB which may be an inefficient use of measurement time, especially at the end of an adaptive track (as the cue tones are always audible) where a bracketing of the target level within a smaller level interval appears feasible. Therefore, we modified the original SIUD procedure by making the level difference changeable, as shown in Figure 3B. If the response was two or zero, the levels of the two tones decreased or increased with a certain step size, respectively, and the level difference remained unchanged. However, if the response indicated 1 tone (indicating that the threshold was bracketed by both presentation levels), a reduced level interval was applied to the two tones, and both tones were concurrently adjusted in level with a given step size.

As mentioned above, the starting level of the probe tone was drawn from a discrete uniform distribution ranging between 35 and 45 dB with a spacing of 1 dB (11 values), while the starting level of the cue tone was 10 dB higher than the probe tone. The level difference between the cue and probe tone was halved to 5 dB when participants first reported that they only heard one tone, and then the level was reduced to 2 dB after the second time they reported that they only heard one tone. The initial step size of the GRaBr procedure for the cue tone was 8 dB, reduced to 6 dB after the first reversal, halved to 3 dB after the second reversal, and eventually to 1 dB after the third reversal, which follows the recommendations by Lecluyse and Meddis (2009).

#### APTA

Figure 3C depicts one run of the APTA procedure. The level kept increasing until the first “yes” response was detected. The level was reset to −10 dB and kept increasing until the second “yes” response was given. Afterward, the level was chosen to be 5 dB lower than the level at the second “yes” response. A run terminated if at least 7 “yes” responses were detected and the maximum level deviation of the last two “yes” responses was less than 3 dB. The threshold was chosen as the sound level of the last “yes” response, plotted with a green line in Figure 3C.

#### QUEST+

QUEST+ is a generalization of the original QUEST procedure for threshold measurement, shown in Figure 3D (Watson, 2017; Watson & Pelli, 1983). In this study, the yes-no task was used for the QUEST+ procedure. The parameter space and stimulus space to initialize QUEST+ were reported in Table 1. L_50_ was set up as a free parameter to be estimated in the range between −10 and 50 dB.

#### MLP

Green (1990, 1993) designed a procedure based on maximum likelihood estimation (MLP) to measure the hearing threshold in a yes-no task, shown in Figure 3E. Following the suggestion of Grassi and Soranzo (2009), the optimal p-target (also corresponding to the sweet point in Brand and Kollmeier (2002) and Green (1995)) was adopted to be 0.6310 in the current study and p_min_ (also referred to as the false-alarm proportion in Green (1995)) varied from 0 to 0.1, with a step size of 0.05 (three values). The range of hypothetical midpoints (i.e., L_50_) of the psychometric function was defined as −10 to 50 dB (choice of stimulus space as required for the procedure, cf. Table 1).

#### UML

As is shown in Figure 3F, the sixth adaptive procedure is UML, which is an extension of the MLP method (Shen & Richards, 2012). A two-down one-up sweet point selection rule was employed. The sweet point is defined as the point at the most informative level of the underlying psychometric function (Lecluyse & Meddis, 2009). Presenting the stimulus at the sweet point would minimize the variability in the threshold estimate (Shen & Richards, 2012). By the end of each track, the mean of the posterior parameter distribution (see Shen & Richards (2012) for details) was applied to estimate the L_50_. The configurations of UML are reported in Table 1.

#### SIAM

Kaernbach (1990) described the unbiased adaptive SIAM procedure which is based on a yes-no task to measure the tone detection threshold, demonstrated in Figure 3G. For each presentation, there was a 50% chance of containing a tone, and the participants were required to answer whether they could hear a tone or not. Seventy-five percent correct tone detection (denoted as “t” in Table 1) was used in the SIAM procedure. The initial step size of the SIAM procedure was 4 dB, halved after the second reversal, and eventually reduced to 1 dB onward after the third reversal.

### Computer Simulations

All algorithms and simulations were developed in MATLAB R2021a (The MathWorks, Inc., Natick, MA, USA) and Octave 5.2.0. The Matlab implementations of the four model-based procedures, i.e., QUEST+, MLP, UML, and SIAMwere provided by Jones (2018), Grassi and Soranzo (2009), Shen et al. (2015), and Schädler et al. (2020), respectively. We implemented the SIUD procedure while Hörzentrum Oldenburg gGmbH provided the Matlab toolbox for the APTA procedure.

A total of 1000 Monte-Carlo simulations were utilized as a numerical method to randomly produce a number of events (here: simulated adaptive tracks) and estimate the underlying parameters of these events (i.e., the average outcome of a given procedure, its standard deviation, and convergence rate). One thousand Monte-Carlo runs were simulated for each simulated listener and each false alarm rate. Monte-Carlo simulations have already been applied to compare different adaptive procedures in many earlier studies (e.g., Hall, 1981; Herbert et al., 2022).

### Evaluation

#### Robustness Bias

To assess the robustness of adaptive procedures, the bias (also known as the signed difference) between the threshold estimates $\hat{L_{50}}$ and the true hearing threshold L_50_ in the k_th_ simulation is calculated in Equation (4):

(4) $$\mathrm{Bias}(k)\;=\;\hat{L_{50,k}}-\;L_{50}$$

The true threshold L_50_ is determined by the level at the center of the range for the psychometric function (Lecluyse & Meddis, 2009). A positive bias indicates that the true threshold is overestimated while a negative bias implies an underestimation of the true threshold.

##### Root mean square error

We calculated the root-mean-square error (RMSE) to examine the robustness of different adaptive procedures under different conditions, using the following formula:

(5) $$\mathrm{RMSE}\;=\;\sqrt{\overset{N}{\underset{k\;=\;1}{\sum }}\frac{{\left(\hat{L_{50,k}}-\;L_{50}\right)}^{2}}{N}}\;$$

N is the number of simulations. RMSE is always greater than or equal to zero, and a larger RMSE indicates a worse performance of the procedure. To estimate the mean and standard deviation of RMSE and conduct the t and ANOVA tests on RMSE, we performed bootstrapping by drawing samples on the estimated threshold 1,000 times (i.e., 1,000 bootstrap replicates) with replacement, where each sample contained *N* = 10,000 data points.

#### Efficiency

##### Normalized efficiency

Taylor and Creelman (1967) proposed the sweat factor as an efficiency index. The sweat factor was widely adopted to compare adaptive procedures that had different rules and number of trials (Amitay et al., 2006; Audiffren & Bresciani, 2022; Leek, 2001; Saberi & Green, 1997; Treutwein, 1995). The empirical sweat factor ${\mathrm{SF}}_{\mathrm{emp}}\;$ is defined as the product of the number of trials (both catch trials and intervals are not included in determining the number of trials N) and the variance of the threshold estimate derived from these trials, expressed by the formula:

(6) $${\mathrm{SF}}_{\mathrm{emp}}\;={\;N\sigma }_{L_{50}}^{2}$$

N denotes the number of trials and $\sigma_{L_{50}}$ the standard deviation of the threshold estimate (how $\sigma_{L_{50}}$ is calculated is given in Equation (8)). We normalized the SF_emp_ by introducing the normalized efficiency (NE):

(7) $$\mathrm{NE}\;=\;\frac{1-p_{\mathrm{aborted}}}{{\tau N\sigma }_{L_{50}}^{2}}$$

p_aborted_ is defined as the percent of the tracks that were aborted during the Monte-Carlo simulations. Only those methods that employ catch trials (i.e., SIUD and GRaBr) were characterized by a non-zero p_aborted_. For all other methods p_aborted_ was set to 0. Furthermore, the time consumption index τ indicates the time approximately consumed for each trial which is set to 1.5 for the one-interval procedures (i.e., SIAM, APTA, QUEST+, MLP, and UML) while 2.5 is assumed for the two-tone procedures (i.e., SIUD and GRaBr). Here, we assume that the duration of one tone is 0.5 s while the response time is 1 s. Hence, a one-interval procedure would require 1.5 s in total for the time consumption index τ. In addition, the pause between two tones is 0.5 s. Therefore, two-tone procedures would need 2 × 0.5 s (duration of one tone) + 0.5 s (pause) + 1 s (response time) = 2.5 s. This reflects the fact that the response interval is usually much smaller than the stimulus presentation time (including pauses before stimulus onset).

##### Rate of convergence

Following earlier studies (Brand & Kollmeier, 2002; Kollmeier et al., 1988; Shen & Richards, 2012), we plotted the standard deviation of threshold estimates $\sigma_{L_{50}}$ to compare the rate of convergence, where the standard deviation at the i_th_ trial $\sigma_{L_{50}}(i)$ is given by:

(8) $$\;\;\sigma_{L_{50}}(i)\;=\sqrt{\frac{1}{N-1}\overset{N}{\underset{k\;=1}{\sum }}\;{\left(\hat{L_{50,k,i}}-\;\mu \right)}^{2}}\;$$

where μ is the mean of the threshold estimates and N is the number of simulations. The “Tidyverse” package (Wickham et al., 2019) developed in R (R Foundation for Statistical Computing) was employed for the statistical analysis of the ANOVA and the post-hoc *t*-test. Four different four-factor ANOVAs were conducted to examine the effect of (a) type of inattention (two levels: long-term/short-term), (b) degree of inattention (fully-/moderately-/non-concentrated), (c) level of false alarm rate (i.e., 0, 0.05, and 0.1), and (d) adaptive procedures on four dependent variables (i.e., bias, root mean square error, normalized efficiency, and standard deviation of threshold estimates).

## Results

### Robustness

#### Bias

**Figure 4.**
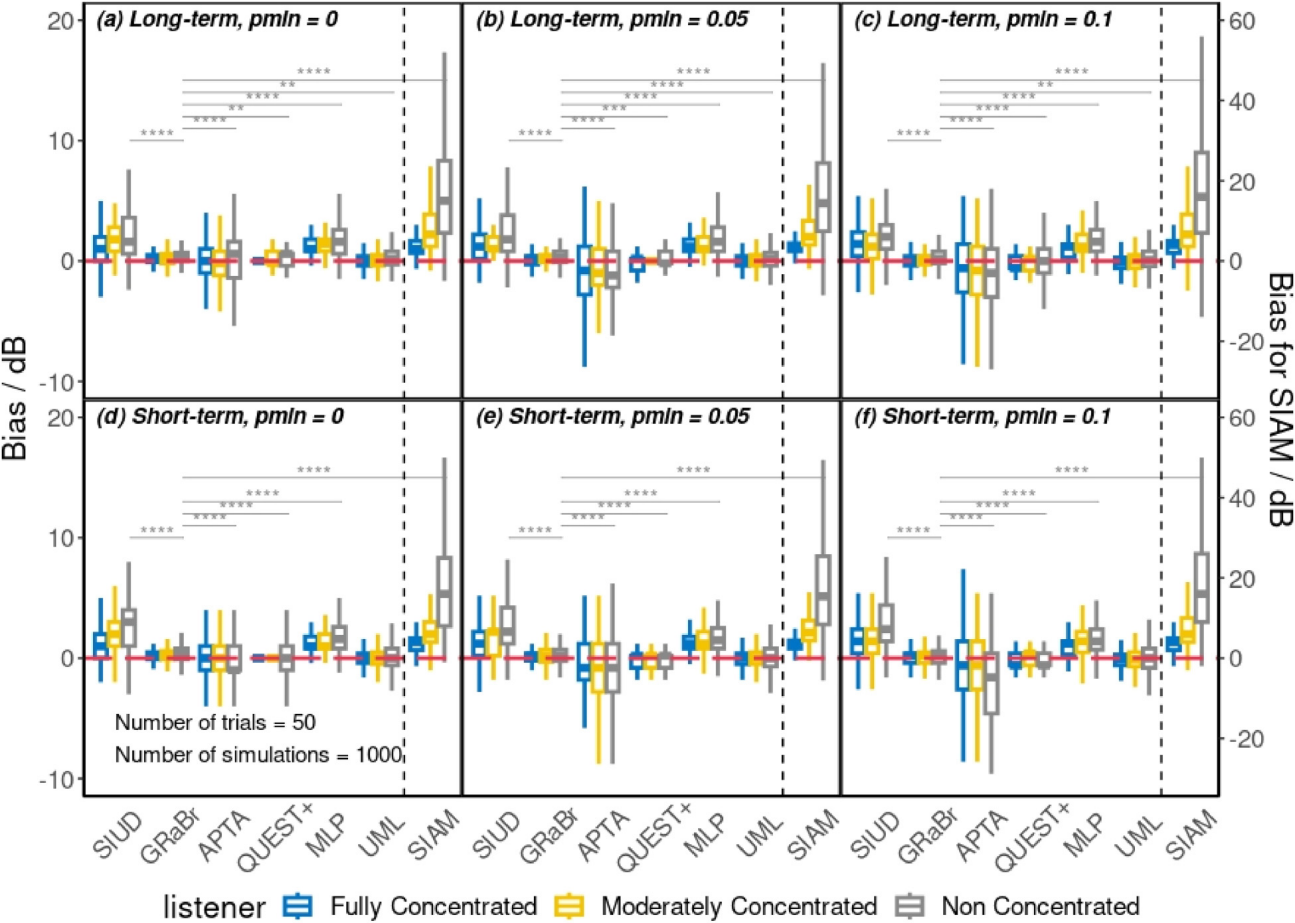
Bias of the threshold estimates $\hat{L_{50}}$ grouped by three simulated listeners (fully-, moderately-, and non-concentrated listeners) across seven adaptive methods (SIUD, GRaBr, APTA, QUEST+, MLP, UML, and SIAM) for the long- and short-term inattentive listener with three different false alarm rates p_min_. Note that the bias of SIAM is plotted with a different scale (given on the right side of the figure) since a scaling factor of 1/3 had to be applied to display the data in the same plot as other procedures. See Figure 3 for an explanation of the abbreviations of the adaptive procedures. Threshold estimation was compared after 50 trials to allow for a fair comparison. Dashed reference line: 0 dB. Median, 25th and 75th percentiles, and interquartile ranges (IQR) are represented in bar-and-whisker plots. The ends of the whiskers describe values within 1.5 × IQR of the 25th and 75th percentiles. The statistical outcome of the pair-wise comparison against GRaBr for the NC listener is visualized via grey solid lines. The level of significance for *p* values is labeled with stars above the lines. Only comparisons that are statistically significant are depicted.

Figure 4 shows the bias of the threshold estimates $\hat{L_{50}}$ for seven adaptive procedures grouped by three simulated listeners for both the long- and short-term inattentive listener with three levels of false alarm rates (0, 0.05, and 0.1). The upper and bottom rows in Figure 4 depict the results of the long- and short-term inattentive listeners, respectively. First, the performance in terms of bias of the long-term inattentive listener was roughly comparable to the short-term inattentive listener as the miss rate of the long-term inattentive listener was aligned with the short-term inattentive listener despite some mismatches in false alarm rate, as shown in Figure 2. Second, as expected, the FC listener was the least biased while the NC listener was the most biased among the three inattentive listeners. As the level of inattention increased (i.e., from the FC to the NC listener), in most cases, the bias increased. GRaBr appeared to be less influenced by the level of inattention than the other procedures whereas SIAM was more influenced by the level of inattention. Third, as the false alarm rate increased, the median bias for most adaptive approaches increased. In general, the median biases of GRaBr, QUEST+, and UML were relatively close to 0 while the median biases of the other adaptive procedures substantially deviated from 0. Moreover, a negative median bias (i.e., an underestimation) was likely to occur if the false alarm rate increased from 0 to 0.1, especially for APTA. Overall, it is evident that an increase in the level of inattention and false alarm rate results in a larger bias and, therefore, worse performance for all adaptive procedures.

The main effect of all four factors (i.e., type and degree of inattention, false alarm rate, and adaptive procedure) was statistically significant, revealed by a four-way ANOVA test (*p *< .05). Subsequently, a pair-wise *t*-test with Bonferroni correction was carried out to compare the bias of adaptive procedures for the long- and short-term inattentive listeners over three simulated listeners with three different false alarm rates. Overall, most adaptive procedures significantly differed from each other in terms of bias (*p* < .05). However, there was one exception: for the long-term MC listener and the short-term NC listener, GRaBr did not differ from UML for all false alarm rates. The complete statistical comparisons are provided in the supplementary document (see Tables S1, S2, and S11 in the online supplemental materials).

#### Root-Mean-Square Error (RMSE)

Comparisons across seven adaptive procedures in terms of RMSE are reported in Table 2. It is apparent that GRaBr produced the smallest mean RMSE among all procedures in all conditions whereas APTA and SIAM had relatively large mean RMSE values. Similar to the results reported above, RMSE increased in case the level of inattention or the level of false alarm rate increased. Generally, the RMSE values of the long-term inattentive listener were comparable to the short-term inattentive listener.

**Table 2.** Mean and Standard Deviation (Mean ± SD) of the Root Mean Square Error (RMSE) for the Seven Adaptive Procedures. The Smaller the RMSE, the More Robust the Procedure Is.

		p_min_	SIUD	GRaBr	APTA	QUEST+	MLP	UML	SIAM
Long-term	FC	0	1.7 ± 0.1	**0.5 ** **± ** **0.0**	2.1 ± 0.2	0.7 ± 0.0	1.4 ± 0.1	0.6 ± 0.0	3.8 ± 0.2
		0.05	1.8 ± 0.2	**0.6 **± **0.0**	2.7 ± 0.3	0.7 ± 0.1	1.4 ± 0.1	0.7 ± 0.1	3.9 ± 0.2
		0.1	1.8 ± 0.1	**0.6 **± **0.0**	3.9 ± 0.5	1.0 ± 0.2	1.4 ± 0.1	0.9 ± 0.2	4.0 ± 0.2
	MC	0	2.5 ± 0.3	**0.6 **± **0.0**	2.2 ± 0.2	0.7 ± 0.1	4.1 ± 1.0	1.0 ± 0.4	11.5 ± 1.2
		0.05	2.7 ± 0.6	**0.6 **± **0.0**	2.7 ± 0.3	0.8 ± 0.1	4.2 ± 1.0	1.3 ± 0.5	11.0 ± 1.3
		0.1	2.8 ± 0.6	**0.6 **± **0.1**	3.8 ± 0.5	1.0 ± 0.2	4.3 ± 0.9	1.5 ± 0.5	12.2 ± 1.3
	NC	0	4.4 ± 0.8	**0.7 **± **0.1**	2.3 ± 0.2	1.0 ± 0.2	7.1 ± 1.2	2.4 ± 0.8	21.0 ± 1.5
		0.05	4.4 ± 0.7	**0.7 **± **0.1**	2.9 ± 0.3	1.1 ± 0.3	7.3 ± 1.1	3.0 ± 0.7	21.3 ± 1.6
		0.1	4.3 ± 0.8	**0.7 **± **0.1**	4.0 ± 0.6	1.4 ± 0.3	6.8 ± 1.0	2.9 ± 0.7	22.0 ± 1.5
Short-term	FC	0	1.8 ± 0.2	**0.5 **± **0.0**	2.1 ± 0.2	0.7 ± 0.1	1.3 ± 0.1	0.6 ± 0.0	4.0 ± 0.2
		0.05	1.8 ± 0.1	**0.5 **± **0.0**	2.7 ± 0.3	0.7 ± 0.1	1.4 ± 0.1	0.7 ± 0.1	3.9 ± 0.2
		0.1	1.7 ± 0.1	**0.6 **± **0.0**	3.7 ± 0.5	1.0 ± 0.2	1.5 ± 0.2	1.0 ± 0.3	4.1 ± 0.2
	MC	0	2.9 ± 0.6	**0.7 **± **0.1**	2.2 ± 0.2	0.8 ± 0.1	4.3 ± 0.9	0.9 ± 0.3	9.5 ± 0.9
		0.05	3.1 ± 0.6	**0.7 **± **0.1**	3.1 ± 0.4	0.9 ± 0.1	4.4 ± 0.9	1.0 ± 0.2	9.7 ± 1.0
		0.1	2.9 ± 0.5	**0.7 **± **0.1**	5.0 ± 0.9	1.3 ± 0.3	3.8 ± 0.9	1.6 ± 0.4	9.7 ± 0.9
	NC	0	4.8 ± 1.1	**0.8 **± **0.1**	2.7 ± 0.4	1.1 ± 0.3	7.4 ± 1.1	2.7 ± 0.7	20.7 ± 1.3
		0.05	5.1 ± 1.0	**0.9 **± **0.1**	4.7 ± 0.9	1.5 ± 0.3	7.3 ± 1.2	2.9 ± 0.7	20.7 ± 1.3
		0.1	5.9 ± 1.1	**0.9 **± **0.2**	9.1 ± 1.4	2.0 ± 0.4	7.1 ± 1.2	3.1 ± 0.6	21.1 ± 1.3

*Note*. Refer to Figure 3 for an explanation of the abbreviations for the adaptive procedure. The smallest mean RMSE value of each simulated listener (rows) is emphasized in bold.

All four factors (i.e., type of inattention, level of inattention, level of false alarm rate, and adaptive procedure) were significant on RMSE, revealed by a four-factor ANOVA test (*p* < .05). The pairwise *t*-tests revealed that the RMSEs of most adaptive procedures significantly differed from each other (*p* < .05). There was no significant difference between SIUD and APTA for the MC listener if p_min_ was 0.05. APTA did not differ from UML for the short-term MC listener if p_min_ was 0. All other pairs were tested to be significantly different (see Tables S3, S4, and S12 in the online supplemental materials for the complete statistical results).

### Efficiency

#### Normalized Efficiency

**Figure 5.**
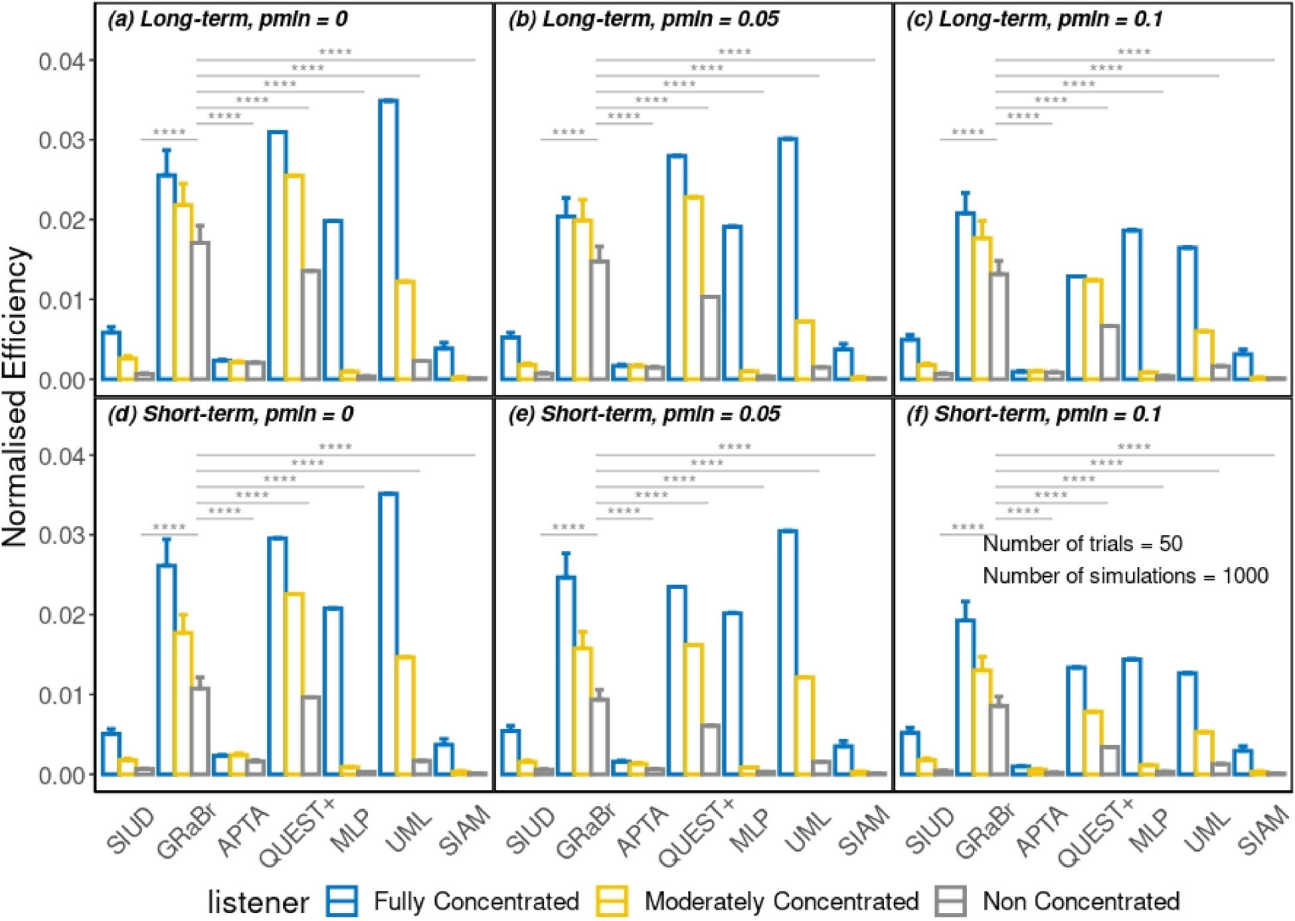
Mean and SD of the normalized efficiency grouped by three levels of inattention across seven adaptive methods for two types of inattentive listeners with three levels of false alarm rate. Only statistical results of the pair-wise comparison against GRaBr for the NC listener (gray solid lines) are plotted. The higher the normalized efficiency, the more efficient the procedure is. Only comparisons that are statistically significant are depicted by grey lines and respective levels of significance. See Figure 3 for an explanation of the abbreviations. Some error bars are so small that they are nearly invisible.

Figure 5 shows the results of the comparison across seven adaptive methods in terms of the normalized efficiency. Overall, adaptive procedures, e.g., GRaBr, QUEST+, and UML exhibited a high normalized efficiency while SIUD, SIAM, and APTA produced a relatively low normalized efficiency. Moreover, the FC listener was the most efficient among the three simulated listeners. Additionally, the lower the false alarm rate, the more efficient the adaptive procedure was.

The main effect of four factors, i.e., type of inattention, degree of inattention, false alarm rate, and adaptive procedure on the normalized efficiency was accessed via a four-way ANOVA test. All four factors produced a significant main effect (*p* < .05). The influence of adaptive procedure on the normalized efficiency over long- and short-term inattentive listeners for FC, MC, and NC simulated listeners with three levels of false alarm rate was investigated employing a pair-wise *t*-test. The results (see supplementary material in Tables S5, S6, and S13 in the online supplemental materials for the complete statistical outcome) revealed that there was a significant difference in the normalized efficiency between adaptive procedures for all simulated listeners, all inattentiveness types, and all false alarm rates (*p* < .05). Compared with the original SIUD procedure, the normalized efficiencies of the GRaBr procedure were significantly higher (*p* < .05), indicating that the modification of the GRaBr procedure with respect to the original SIUD procedure shows a positive effect. Finally, UML exhibited a significantly higher normalized efficiency than the baseline MLP procedure (*p < *.05).

#### Rate of Convergence

**Figure 6.**
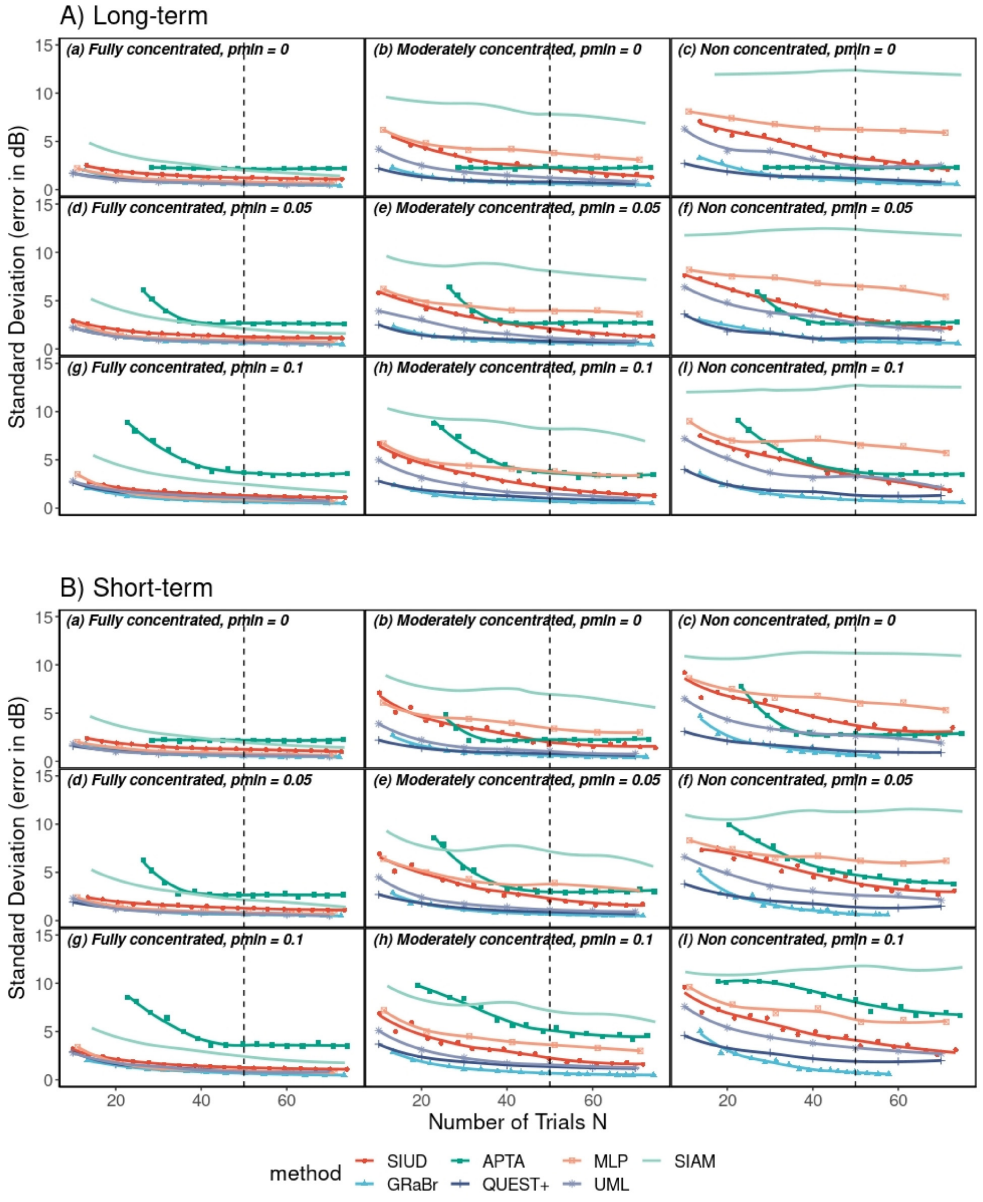
Standard deviation $\sigma_{L_{50}}$ of threshold estimates, $\hat{L_{50}}$ as a function of the number of trials for seven adaptive procedures. Vertical dashed line: number of trials employed in Figures 4 and 5 and Table 2. See Figure 3 for an explanation of the abbreviations for adaptive procedures.

The standard deviation $\;\sigma_{L_{50}}$ of threshold estimates, plotted as a function of the number of trials for seven adaptive procedures, is shown in Figure 6. The results of the long- and short-term inattentive listener are presented in Figure 6A and B, respectively. The standard deviations monotonically decreased as the number of trials increased for most adaptive procedures. Therefore, most adaptive procedures converged. On the contrary, no clear convergence was observed for SIAM if the listener was not fully concentrated. GRaBr and QUEST+ exhibited considerably lower standard deviations for a given number of trials in most conditions. GRaBr produced lower standard deviations than the baseline SIUD procedure while UML yielded lower standard deviations than the original MLP procedure. Increasing the level of inattention or false alarm rate generally led to an elevated standard deviation. In other words, an adaptive procedure converged more quickly for the FC listener with a lower false alarm rate than the NC listener with a higher false alarm rate.

An ANOVA test implied that the main effect of all four factors (i.e., type and degree of inattention, false alarm rate, and adaptive procedure) on the average standard deviation $\;\sigma_{L_{50}}$ cross-trials was significant (*p* < .05). The effect of adaptive procedures on $\sigma_{L_{50}}$ for the long- and short-term inattentive listener over three simulated listeners with three false alarm rates was assessed via pair-wise *t*-test. As expected, most of the adaptive procedures did not differ from each other for the FC listener, indicating that all adaptive procedures were similarly efficient for the ideal listener. However, most adaptive procedures significantly differed from each other for the MC and NC listeners with several exceptions: GRaBr did not differ from UML and QUEST+, and there was no significant difference between GRaBr and APTA for the NC listener in case p_min_ was 0. See the supplementary material in Tables S7, S8, and S14 in the online supplemental materials for the complete statistical results.

## Discussion

We evaluated seven adaptive procedures (three model-free and four model-based), in terms of robustness against inattention and efficiency, by Monte Carlo simulations. Two inattention models were employed for this purpose, termed long- and short-term inattention, where the level of inattention and the false alarm rate varied. Some of the well-established procedures (i.e., APTA representing the standard clinical procedure, SIUD, SIAM, and MLP) exhibited surprisingly little robustness against inattention and a considerable drop in efficiency even with moderate levels of long- and short-term inattention. The proposed procedure GRaBr, on the other hand, was rather accurate and robust for threshold measurements, revealed by a small bias and RMSE of threshold estimates against the “true” threshold. GRaBr also yielded considerable efficiency for all levels of inattention, indicated by the normalized efficiency index and rate of convergence. GRaBr outperformed the baseline SIUD while its performance was comparable with the state-of-the-art model-based procedure, i.e., QUEST+.

### Adaptive Procedures

#### APTA

 For a false alarm rate of p_min _= 0, the median estimated thresholds of the APTA procedure are close to the “true” threshold for the long- and short-term FC, MC, and NC listeners, thus the APTA procedure appears to be an unbiased estimation procedure for all types of inattentive observers with a low false alarm rate. These simulation results are consistent with human experiments conducted by Swanepoel et al. (2010) who used automatic audiometry using smartphones and compared the results with manual audiometry. There was no significant difference between automatic and manual audiometry, indicating that the APTA procedure was unbiased and robust. Moreover, Guo et al. (2021) evaluated the accuracy of the automatic audiometry application using easily accessible true wireless stereo earbuds. The verification experiment suggested that the APTA procedure was accurate enough for the threshold measurement. However, if p_min_ is larger than or equal to 0.05, the threshold estimate is no longer accurate. Therefore, experimenters should choose APTA carefully when measuring audiograms and ensure that the participants make confident decisions as much as they can.

If both the false alarm rate and the miss rate are low, the standard deviation $\sigma_{L_{50}}\;$ the APTA procedure is overall comparable to the other adaptive procedures, thus yielding APTA to be comparatively efficient. This is in line with the findings of Swanepoel et al. (2010) who examined the efficiency of the APTA procedure under the restriction of a limited measurement time (i.e., an average of 7.2–7.7 min for both ears of normal hearing subjects across seven frequencies which corresponds to approximately 24 trials per adaptive track). However, APTA might have a convergence problem for participants who have a large false alarm rate and miss rate: in some groups, e.g., the short-term NC listener with a 0.1 false alarm rate, the shallow slope of the decrease in standard deviation across the number of trials (cf. Figure 6) indicates that the precision increases less than expected from the 1/ $\sqrt{N}$ -law for independent estimations in each trial. This lack of convergence might be due to the accumulation of inconsistent trials across the whole measurement track that all add up to losing the correct target level orientation. As a consequence, it is advisable to supervise the participants to maintain a low level of false alarm rate and miss rate when performing the APTA procedure. Otherwise, the adaptive track might not be efficient.

#### QUEST+

Generally, the QUEST+ procedure could estimate thresholds both accurately and efficiently even for different degrees of inattention and false alarm rates, which is in line with previous studies, e.g., Audiffren and Bresciani (2022). Our present study mainly adjusts the miss rate of the logistic psychometric function to model long-term inattention. It is not surprising that QUEST+ handles such an inattention model well since it considers the influence of the miss rate and could even estimate the miss rate at the end of a track. However, as Audiffren and Bresciani (2022) explain, the QUEST+ procedure would no longer be robust if the listener behavior was modeled differently (e.g., with a non-logistic psychometric function like a beta distribution that violates the basic assumptions of the QUEST+ procedure). For the short-term inattention listener, an increase in inattention leads to an increase in the variance of threshold estimates. Since the initial PF in the short-term inattention model (indicated in Equation (2)) does not exhibit a fixed and stationary miss rate, this mismatch to the model assumed by the QUEST+ procedure results in a high uncertainty of the threshold estimates. In comparison to GRaBr, this produces a significantly larger RMSE and a lower normalized efficiency at least for the short-term inattentive observer. This suggests that the GRaBr is a better choice than the QUEST+ procedure for unsupervised psychophysical tests, e.g., using a smartphone.

#### MLP and UML

The MLP procedure estimates the hearing threshold precisely for the FC listener, however, it yields severe overestimates for the MC and NC listener. Green (1995) reported that the MLP procedure produced a standard deviation of nearly 5 dB if the N was smaller than 20 trials, and 2.5 dB after 50 trials. Our results are roughly in line with Green (1995). Green (1993) indicated (without proof) that the MLP procedure was more efficient than the SIAM procedure. This was later questioned by Shepherd et al. (2011) who disagreed with Green's statement. In our study, the comparison of the rate of convergence between the SIAM and MLP procedures (cf. Figure 6) indicates that the MLP procedure has a smaller standard deviation for the three simulated listeners given the same number of trials than the SIAM procedure. Hence, our data support Green's (1993) assertion that MLP is more efficient than SIAM. Moreover, for the MC and NC listeners, the normalized efficiency of the procedure is much lower and the bias is larger than for the model-free procedures considered here (e.g., GRaBr). Therefore, the use of the MLP procedure for smartphone experiments is not encouraged since its performance is greatly affected by the status of the listeners.

Several researchers (Green, 1995; Gu & Green, 1994; Lecluyse & Meddis, 2009; Leek, 2001; Leek et al., 2000; Shepherd et al., 2011) highlight that the MLP is not a robust procedure and try to assess why the MLP procedure deviates from the expected advantageous high efficiency and fast convergence. On one hand, Green (1995) assumed that inattentive participants produce unreliable results. In support of that, Gu and Green (1994) reported that inattention occurring in an early trial (especially before the fifth trial) makes the measurement inaccurate. Our simulations show that the inattention had less effect on the threshold estimates when *N* is larger than five trials, indicating that in the adaptive strategy of the MLP procedure, the early trials have more weight/importance than the late trials. Furthermore, Leek et al. (2000) performed human experiments to validate the MLP procedure and found out that it was difficult and costly for listeners to maintain concentration. Leek et al. (2000) also point out that the MLP procedure is inappropriate for tasks in which the listener model is not based on a fixed psychometric function. On the other hand, Lecluyse and Meddis (2009) did not attribute the poor performance of the MLP to inattentive listeners but rather argued that the adaptive procedure itself results in poor performance. They demonstrated that the adaptive strategy of the MLP procedure is somehow self-reinforcing which prevents a regression to the true threshold, leading to permanently false estimates. Lecluyse and Meddis (2009) further suggested that those false estimates do not disappear even if the number of trials becomes larger. Our data support that both Green (1995) and Lecluyse and Meddis (2009) are correct in their statements: while the different inattentive observer models clearly lead to a significant bias and reduced efficiency (in line with Green (1995)), the standard deviation for the SIUD procedure exhibits a much steeper decline with an increasing number of trials for the initial trials than for a larger number of trials (cf. Figure 6) where most other procedures show a steeper slope. This supports the assumption by Lecluyse and Meddis (2009). In conclusion, the low reliability of the MLP procedure appears to originate both from the inattentive participants and the procedure itself.

The optimized procedure UML significantly surpasses the original MLP in terms of robustness and efficiency, which is in agreement with the previous study (Shen & Richards, 2012). Since UML is specially designed to solve the shortcoming of inattention, the better performance of UML is expected. As a consequence, when choosing an adaptive procedure for mobile devices, where listeners are highly likely to be distracted, UML appears to be better suited in comparison to MLP. However, with increasing inattentiveness, the model assumptions are increasingly violated which leads to a decrease in efficiency and a slight increase in bias, especially for the non-stationary inattention case. In comparison to GRaBr, UML shows a poorer performance in these conditions as UML typically does not incorporate the short-term inattention model with the “unusual” psychometric function when designing the adaptive procedure while GRaBr, as a model-free procedure, is relatively less sensitive to the inattention model. Hence, GRaBr appears to be preferable in cases where stable attention of the subject cannot be secured.

#### SIAM

SIAM is considered to be less robust and efficient than the other adaptive procedures in most cases. SIAM was introduced by Kaernbach (1990) who demonstrated that the SIAM procedure was reliable and robust for the FC listener by considering the response bias. Shepherd et al. (2011) reported that the SIAM procedure was efficient because a single interval task is employed which consumes less time per trial than if nIFC procedures are used. However, our simulations indicate that the SIAM procedure yields a large bias for inattentive listeners and is generally not as efficient as other procedures, especially for the groups of MC and NC listeners. The main reason is that the SIAM procedure according to Kaernbach (1990) assumes that both the hit and false-alarm rates assume maximum values of 1 which is reflected in the payoff matrix that controls the adaptive track. This assumption, however, is only true for the FC listener, whereas the maximum hit and false-alarm rates for the MC and NC listener in our inattentive model are reduced to be less than one, i.e., smaller than for the FC listener. If these values are known a priori, a modified payoff matrix might be employed which may avoid the observed bias. However, such a priori knowledge is usually not available during the time of testing. As a consequence, SIAM appears to be a viable procedure for the FC listener in the laboratory but does not appear to be an unbiased, efficient, and robust threshold estimation method as soon as an MC or NC listener is assumed.

#### SIUD and GRaBr

Lecluyse and Meddis (2009) examined the reliability of the SIUD and MLP procedure for the FC listener and found that the threshold estimates did not differ, even though the MLP procedure yielded a larger standard deviation. Our experiments are in agreement with Lecluyse and Meddis (2009) insofar as there is no difference in the threshold estimates between the SIUD and MLP procedures. When considering the bias for the MC and NC listener, the SIUD procedure is more robust than the SIAM procedure and comparable to the MLP procedure whereas the rate of convergence and the normalized efficiency are superior for the SIUD procedure (*p* < .001). This compromised performance of the MLP and SIAM procedures for the MC and NC listener appears to be due to the assumption of a fixed psychometric function during the threshold estimation process which is not met unless for an FC listener as assumed by the procedures. The SIUD procedure, on the other hand, is model-free which helps to overcome the inattention to some extent based on the numerical data.

An efficiency comparison against SIUD with other adaptive procedures (e.g., MLP) was not carried out in Lecluyse and Meddis (2009), which motivated parts of our study. We observe that the MLP procedure is generally less efficient than SIUD for all levels of inattention and false alarm rate which points towards an advantage of the SIUD over the MLP procedure for practical applications where the attentiveness of the listener is not assured.

One difference between the SIUD procedure and the other procedures established so far (e.g., MLP, APTA, SIAM, UML, and QUEST+) is the psychophysical task (0, 1, or 2 tones detected with one of the two tones being presented at a level 10 dB above the other tone) which is intuitive and easy for naïve subjects to perform. Hence, this procedure is attractive for use in smartphone applications which motivated its usage in the GRaBr procedures (see below). Lecluyse and Meddis (2009) pointed out that there is little training effort needed which was supported by Shepherd et al. (2011). Rather, for the single-interval-yes-no task, Gu and Green (1994) showed a significant difference between the naïve and experienced listeners employing the MLP procedure. Leek et al. (2000) further confirmed this finding. Amitay et al. (2006) validated that more trials were required for naïve listeners applying the MLP procedure for threshold assessment since too few trials produced a very large variance.

The GRaBr procedure is very similar to the SIUD procedure during the initial part of the adaptive track. Once the threshold region is approached, however, GRaBr makes the sound level difference between the two tones adaptive with the effect that more cue tones are presented near the threshold in GRaBr than in SIUD (see Figure 3). This provides more information per trial about the psychometric function close to the threshold than if a fixed 10-dB difference is employed. In addition, GRaBr treats the responses gained from presenting each of both tones in a similar way, i.e., the audibility of the cue tone is exploited to steer the adaptive level placement. In contrast, in the SIUD, the audibility of the cue tone is only evaluated in sham trials which causes additional time and effort and hence a reduction in efficiency in comparison to GRaBr. Therefore, the GRaBr procedure was expected to be more efficient than the SIUD procedure. This was confirmed by the normalized efficiency estimated in our simulations (cf. Figure 6).

Choosing model-free non-parametric procedures or model-based parametric procedures is highly dependent on the context and the availability of previous information about the performance of the subjects. If the shape of the psychometric function is roughly known a priori, model-based procedures appear advantageous because they can assess the target threshold more quickly and efficiently than non-parametric procedures. However, estimating or assuming the parameters may be problematic, and any inconsistent response behavior of the subject might lead to “unforgiving” slow convergence and a bias in the resulting threshold estimate. Moreover, inaccurate parameter choices in model-based procedures may produce incorrect estimates (Audiffren & Bresciani, 2022). Hence, model-free procedures are often preferred by experimenters since they usually are insensitive to lapses and to “unusual” shapes of the psychometric functions that might not match the expectation. Moreover, due to their robustness against the slope of the underlying psychometric function, they are also quite independent from the step size chosen (albeit the product of step size and slope of the psychometric function is a scale-invariant parameter which has some effect on the efficiency of the procedures). Hence, in agreement with some previous studies, e.g., Smits et al. (2022), the current study shows that model-free procedures (such as GRaBr) typically do not perform worse than parametric procedures and can yield high robustness against comparatively drastic changes of the psychometric functions due to inattention that are considered here.

Taken together, the GRaBr procedure is recommended for the (hearing) threshold detection with potentially inattentive observers due to its high efficiency and robustness against inattention as well as due to the psychophysical task employed (i.e., the graded response of 0, 1, or 2 tones detected) which is supposed to be easily employed by naïve listeners.

### Influence of Inattention and False Alarm Rate

In our numerical experiments, the impact of three independent factors (i.e., type and level of inattention, and level of false alarm rate) is systematically investigated. Previously, Green (1995) examined the two factors miss rate and false alarm rate on only one model-based procedure MLP and found that both factors impacted the accuracy of MLP. We extended the scope by assessing the influence of inattention and false alarm rates on six additional adaptive procedures including three model-free procedures. Furthermore, we implemented a short-term inattention model, which differs from Green (1995).

#### Level of inattention and false alarm rate

The robustness and efficiency of adaptive procedures tend to diminish with increasing levels of inattention or false alarm rates, as evidenced by our simulations (e.g., Figures 4 and 5). To mitigate these effects, it is essential for experimenters to monitor and ensure participant attention, as highlighted by Leek et al. (2000), and to maintain a low false alarm rate. In scenarios where distractions or high false alarm rates are anticipated, or where close supervision is impractical—such as in smartphone-based remote hearing assessments—consideration should be given to adaptive procedures that are less sensitive to these factors, like GRaBr, or to the use of model-free approaches.

#### Type of inattention

A short-term inattention model is introduced that is motivated by the possible distraction from external events. It differs from the well-known long-term inattention model of sustained inattention proposed by Green (1995). This short-term inattention model has a comparable effect on adaptive procedures to the long-term inattention model at the same level of inattention, which is expected. While Green (1995) mainly models the inattention process on the overall psychometric function, we, however, explicitly model the inattention for a single trial and focus on the research question of whether different procedures are differently sensitive to this “unusual,” newly introduced single-trial-PF. Please note that in the long-term inattention model, p(“yes”|inattention) is fixed at p_min_. However, more generally, p(“yes”|inattention) can be assigned to any probability in the short-term inattention model, allowing greater flexibility in characterizing inattention events.

A direct comparison of the effect of both types of inattention models on the different adaptive procedures is difficult since the same parameter values in both models lead to slightly different long-term psychometric functions (see Figure 2). Table 3 therefore compares the normalized efficiency values from Figure 5 for those parameter combinations that exhibit the same equivalent expected PF for the short-term model with the respective PF of the long-term model (see the section Methods - Inattention model). Even though the differences for the various adaptive procedures are small, there are some statistically significant differences in the normalized efficiency between the long- and short-term inattention model for most adaptive procedures (*p* < .0001), indicating that the short-term inattention has a larger negative impact on the performance of the procedures (e.g., MLP) if compared on the bases of the same “effective” psychometric function.

**Table 3.** Comparison of the Normalized Efficiency Between the Long-Term Inattention Model and the Corresponding (Comparable) Short-Term Inattention Model (Derived Given the Equivalent Expected PF in Equation (3)) in Terms of *t* Value and the Level of Significance for *p* Value, Implied Via *t* Tests.

		p_min_	SIUD	GRaBr	APTA	QUEST+	MLP	UML	SIAM
MC	Long-term	0.05	0.0ns	0.2****	−0.9****	0.2****	2.3****	−14.5****	−0.4****
	Short-term	0							
MC	Long-term	0.1	0.2****	0.2****	−0.7****	−5.9****	0.5****	−14.4****	−0.5****
	Shot-term	0.05							
NC	Long-term	0.1	0.1****	0.3****	−1.4****	−8.1****	4.4****	−0.8****	−0.1****
	Short-term	0							

#### Structural stability of tracking procedures

The tracking procedures considered here differ in their stability against non-stationary lapses of attention. It may be interesting to investigate in more detail how sensitive the adaptive track is to an incidence of inattention during the trials immediately following the incidence, and how many trials it would take for the adaptive track to return to the neighborhood of the ground-truth threshold. Note, however, that a detailed micro-analysis of all procedures employed that would uncover the exact mathematical reason for the (in)stability of the respective tracking method is beyond the scope of the current paper. One way to achieve this might be to model the tracking procedures as Markov chains (e.g., Kollmeier et al., 1988), where the dynamics of the flow of level distributions across trials may be analyzed by eigenvalues of the respective transition matrices.

### Limitations

One possible limitation of the current study is that we only set up the true threshold to a single value, i.e., 15 dB in combination with a fixed slope of the psychometric function and a uniform distribution of starting levels (range of 10 dB) that all approximate a realistic experiment with human observers. Even though these parameters are highly interrelated and are expected to have only a marginal effect on the main outcomes of our study (see below), a larger variation of these parameters might be considered in future studies, e.g., randomly drawn threshold and slope parameters could be considered (Shen & Richards, 2012). This might also avoid possible misjudgments about the value of adaptively fixating the step size: If the distribution of initial levels (in relation to the distance from the true threshold) is too narrow, it could happen that procedures that adaptively determine the step size would perform very differently from procedures with pre-specified step sizes because the initial trials of a track would exhibit too little variations across repetitions of the Monte-Carlo simulations.

However, within the given numerical limits the simulations are assumed to be shift-invariant with respect to the true threshold and scale-invariant with respect to the product of the (initial) step size of the respective procedures and the slope of the underlying psychometric function. Hence, even if threshold and slope parameters are changed, the simulation results will not change as long as these invariants are still in place. A change in the initial step size and in the width of the distribution of initial levels would therefore be the only parameters that will produce a slight, but notable change in the simulation results. Please note that the effects on initial trials may require further investigation, as the ultimate goal of this procedure is to determine thresholds using a minimum number of trials. As they only have an impact on the first few trials of the simulation, the main outcomes of the simulations are expected to be unchanged. This assumption is based on findings by Kollmeier et al. (1988), which demonstrate that the influence of the starting parameters on the distribution of levels in an adaptive track vanishes quickly, especially after the first reversal in the track. Hence, a systematic variation of the starting level parameters is expected to yield too few effects to be of interest in the current study, given the already large number of parameters and versions that are being reported on in the current study.

A similar argument holds for the number of trials which is restricted to 50 in our study. It could be expanded to larger values (e.g., 100 and 200 as in Audiffren & Bresciani, 2022). However, the convergence of the procedures considered here was already observed for the 50 trials such that no new information is expected for longer runs. In addition, in practical experiments, the limited measurement time should be distributed to more, but shorter tracks rather than to fewer, but longer tracks in order to average out individual track-to-track variability of the “true” threshold (Kollmeier et al., 1988).

Finally, the simulations employed and discussed here need to be supplemented by experimental data with real human subjects preferably with a variation of the type and level of inattention to validate the simulations performed here. Even though it is difficult to quantify inattention in daily life, future studies will have to systematically study the effect of limited cognitive resources (including attention) on the outcome of psychophysical experiments.

## Conclusion

Inattentiveness of the observer—simulated here with a long- and short-term inattentive behavior model and a moderately- and non-concentrated observer in comparison to a fully concentrated observer—exhibits a major influence on the robustness and the efficiency of the various adaptive psychoacoustic procedures employed here. Most of these have been well-established for well-controlled laboratory conditions in the past. As a consequence, adaptive tracking procedures that have been validated in laboratory studies for the fully concentrated observer cannot be simply transferred to non-laboratory situations with several possible sources of distraction, e.g., smartphone experiments in the real world.

The short-term inattentive observer—which has been introduced here to reflect typical disturbances during real-life conditions using smartphones as a measurement tool—provides a significantly different challenge for the robustness and efficiency of the adaptive psychophysical tracking methods studied here to the well-known long-term inattentive observer if compared at the same rate of inattention or the same shape of the long-term psychometric function. In addition, the false alarm rate significantly influences the robustness and efficiency of adaptive procedures. Generally, as the false alarm rate increases, both robustness and efficiency decrease for most adaptive procedures.

The different psychophysical adaptive tracking methods vary considerably with respect to their robustness against inattentiveness. Overall, the newly introduced method GRaBr optimizes the baseline method SIUD and shows at least comparable performance with some of the latest model-based adaptive procedures, e.g., QUEST+. GRaBr provides relatively high normalized efficiency and high robustness against the different conditions of user inattentiveness. This is due to the design of the psychophysical task, which uses a graded response with 0, 1, or 2 tones detected in a trial, and its simple yet “forgiving” tracking algorithm that considers only the most recent response history of the adaptive track. Hence, the GRaBr procedure appears to be recommendable both for well-controlled in-lab hearing assessments and for psychophysical measurements using mobile devices in real life (e.g., smartphones).

## Supplemental Material

sj-docx-1-tia-10.1177_23312165241288051 - Supplemental material for How Does Inattention Influence the Robustness and Efficiency of Adaptive Procedures in the Context of Psychoacoustic Assessments via Smartphone? Supplemental material, sj-docx-1-tia-10.1177_23312165241288051 for How Does Inattention Influence the Robustness and Efficiency of Adaptive Procedures in the Context of Psychoacoustic Assessments via Smartphone? by Chen Xu, David Hülsmeier, Mareike Buhl, and Birger Kollmeier in Trends in Hearing

sj-docx-2-tia-10.1177_23312165241288051 - Supplemental material for How Does Inattention Influence the Robustness and Efficiency of Adaptive Procedures in the Context of Psychoacoustic Assessments via Smartphone? Supplemental material, sj-docx-2-tia-10.1177_23312165241288051 for How Does Inattention Influence the Robustness and Efficiency of Adaptive Procedures in the Context of Psychoacoustic Assessments via Smartphone? by Chen Xu, David Hülsmeier, Mareike Buhl, and Birger Kollmeier in Trends in Hearing

sj-docx-3-tia-10.1177_23312165241288051 - Supplemental material for How Does Inattention Influence the Robustness and Efficiency of Adaptive Procedures in the Context of Psychoacoustic Assessments via Smartphone? Supplemental material, sj-docx-3-tia-10.1177_23312165241288051 for How Does Inattention Influence the Robustness and Efficiency of Adaptive Procedures in the Context of Psychoacoustic Assessments via Smartphone? by Chen Xu, David Hülsmeier, Mareike Buhl, and Birger Kollmeier in Trends in Hearing

## Figures and Tables

**Figure A1.**
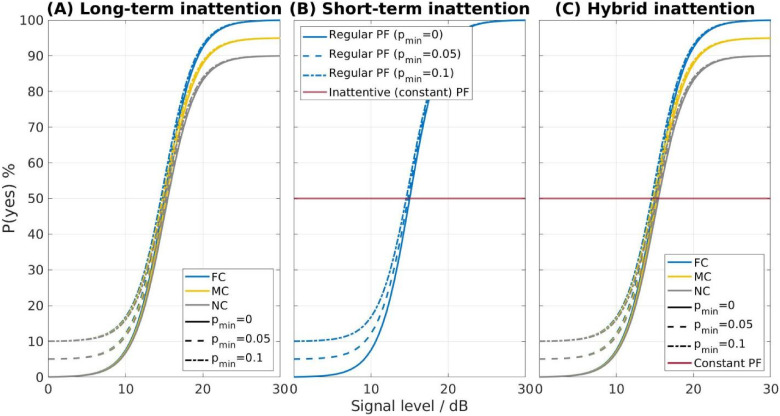
(A) long- and (B) short-term attention model (replicated from Figure 2 in the main corpus for comparison) (C) observer model for the hybrid inattention model (i.e., a listener is distracted both by the long- and short-term inattention simultaneously).

**Figure A2.**
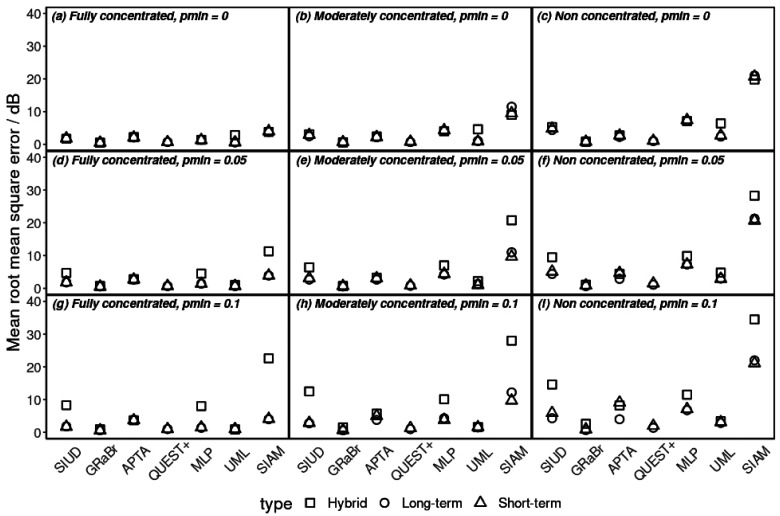
Mean root-mean-square error (RMSE) of the threshold estimate for a track length of 50 for three types of inattentive listeners.

## Appendix: Hybrid Inattention Model

We evaluated a hybrid inattentive listener model that comprises both the long- and short-term inattention model, where the psychometric function is shown in Figure A1(C). Again, for the hybrid inattentive listener, three levels of inattention and three levels of false alarm rate are adjusted so that they can be compared with the other types of inattentive listeners. More specifically, a hybrid FC, MC, and NC listener responds randomly in 0%, 10%, and 20% trials and exhibits a p_max_ of 1, 0.95, and 0.9, respectively. Subsequently, simulations with this model and the seven adaptive procedures were performed. Results are now presented in Figure A2, where different point shapes represent three types of inattentive listeners.

As expected, the hybrid inattentive listener exhibited a larger threshold estimation error than the other two types of inattentive listener, while the main effect of the false-alarm rate and the level of inattention on the performance for the different adaptive procedures stays the same as for the main two listener types. Specifically, our proposed GRaBr procedure yields a relatively small RMSE for all types of inattentive listeners among all adaptive procedures.

## Glossary

AFC: alternative forced choice
ANOVA: analysis of variance
APTA: automated pure-tone audiometry
FC: fully-concentrated
GRaBr: graded response bracketing
MC: moderately-concentrated
MLP: maximum likelihood procedure
NC: non-concentrated
NE: normalized efficiency
nIFC: n-interval forced-choice
PF: psychometric function
RMSE: root mean square error
SIAM: single interval adjustment matrix
SIUD: single interval up and down
UML: updated maximum likelihood
