# Supplementary material for: How Does Inattention Influence the Robustness and Efficiency of Adaptive Procedures in the Context of Psychoacoustic Assessments via Smartphone?
Source: Trends Hear. 2024 Nov 18;28:23312165241288051. doi: 10.1177/23312165241288051 (PMC11574912; doi:10.1177/23312165241288051)
Supplement: sj-docx-1-tia-10.1177_23312165241288051 - Supplemental material for How Does Inattention Influence the Robustness and Efficiency of Adaptive Procedures in the Context of Psychoacoustic Assessments via Smartphone? [file sj-docx-1-tia-10.1177_23312165241288051.docx]

TABLE S1. Statistical analysis of pair-wise t-test (t value and p value significance^a^) on the **bias** of the threshold estimates L_50_ between different adaptive procedures simulated for the fully-, moderately-, and non-concentrated listener, for the **long-term inattentive listener**. The higher the t value is, the larger the difference between the adaptive procedure is. The ‘-’ sign in t value denotes a negative difference. FC: Fully-concentrated listener; MC: Moderately-concentrated listener; NC: Non-concentrated listener. cf. Fig. 3 for an explanation of the abbreviations.

|  |  | FC | | | MC | | | NC | | |
| --- | --- | --- | --- | --- | --- | --- | --- | --- | --- | --- |
| Group I | Group II | p_min_ = 0 | p_min_ = 0.05 | p_min_ = 0.1 | p_min_ = 0 | p_min_ = 0.05 | p_min_ = 0.1 | p_min_ = 0 | p_min_ = 0.05 | p_min_ = 0.1 |
| SIUD | GRaBr | 24.7**** | 25.0**** | 26.3**** | 24.7**** | 20.9**** | 23.2**** | 20.9**** | 22.2**** | 20.5**** |
| SIUD | APTA | 17.3**** | 21.0**** | 23.8**** | 20.6**** | 21.2**** | 24.9**** | 21.5**** | 25.0**** | 25.8**** |
| SIUD | QUEST+ | 27.8**** | 28.4**** | 28.2**** | 27.3**** | 23.3**** | 24.7**** | 21.7**** | 23.0**** | 21.4**** |
| SIUD | UML | 28.0**** | 28.1**** | 28.8**** | 23.8**** | 18.9**** | 21.1**** | 15.2**** | 13.7**** | 13.6**** |
| SIUD | MLP | 2.7ns | 2.7ns | 2.8ns | -2.8ns | -3.5* | -3.4* | -4.4*** | -4.9**** | -4.7**** |
| SIUD | SIAM | -32.2**** | -31.6**** | -30.6**** | -28.8**** | -25.9**** | -26.4**** | -36.0**** | -35.4**** | -37.8**** |
| GRaBr | APTA | 4.4*** | 9.7**** | 14.6**** | 5.3**** | 9.4**** | 14.2**** | 6.5**** | 11.6**** | 15.5**** |
| GRaBr | QUEST+ | 7.0**** | 7.0**** | 7.9**** | 7.1**** | 6.9**** | 6.1**** | 4.0** | 4.3*** | 4.6**** |
| GRaBr | UML | 6.9**** | 6.4**** | 7.7**** | **2.5ns** | **0.9ns** | **2.1ns** | -3.9** | -5.0**** | -4.1** |
| GRaBr | MLP | -30.0**** | -30.8**** | -33.1**** | -15.6**** | -16.8**** | -17.3**** | -17.1**** | -18.0**** | -18.0**** |
| GRaBr | SIAM | -55.5**** | -55.6**** | -53.9**** | -35.8**** | -33.1**** | -33.5**** | -43.6**** | -42.9**** | -45.1**** |
| APTA | QUEST+ | -1.7ns | -7.2**** | -11.8**** | -2.5ns | -6.8**** | -11.8**** | -4.2*** | -9.1**** | -12.9**** |
| APTA | UML | -1.9ns | -7.5**** | -12.2**** | -3.8** | -8.1**** | -12.4**** | -7.5**** | -11.8**** | -15.1**** |
| APTA | MLP | -16.9**** | -20.8**** | -23.5**** | -16.3**** | -19.4**** | -22.6**** | -18.5**** | -21.4**** | -23.7**** |
| APTA | SIAM | -40.3**** | -41.3**** | -39.9**** | -36.0**** | -34.5**** | -36.6**** | -44.2**** | -44.5**** | -47.8**** |
| QUEST+ | UML | -0.3ns | -0.7ns | -0.8ns | -2.8ns | -3.4* | -2.2ns | -5.5**** | -6.6**** | -6.1**** |
| QUEST+ | MLP | -33.2**** | -34.6**** | -32.4**** | -17.2**** | -18.4**** | -18.8**** | -17.8**** | -18.7**** | -18.8**** |
| QUEST+ | SIAM | -57.2**** | -57.7**** | -54.0**** | -36.6**** | -33.9**** | -34.2**** | -43.9**** | -43.2**** | -45.4**** |
| UML | MLP | -33.6**** | -34.5**** | -33.8**** | -15.9**** | -16.3**** | -17.1**** | -14.6**** | -14.1**** | -14.4**** |
| UML | SIAM | -57.4**** | -57.6**** | -54.8**** | -36.0**** | -32.8**** | -33.5**** | -42.1**** | -40.6**** | -43.1**** |
| MLP | SIAM | -37.6**** | -37.3**** | -35.9**** | -25.2**** | -22.5**** | -23.1**** | -31.2**** | -30.4**** | -33.2**** |

^a^ ns: not significant; *p<0.05; **p<0.01; ***p<0.001; ****p<0.0001

TABLE S2. Statistical analysis of pair-wise t-test (t value and p value significance^a^) on the **bias** of the threshold estimates L_50_ between different adaptive procedures simulated for the fully-, moderately-, and non-concentrated listener, for the **short-term inattentive listener**. The higher the t value is, the larger the difference between the adaptive procedure is. The ‘-’ sign in t value denotes a negative difference. FC: Fully-concentrated listener; MC: Moderately-concentrated listener; NC: Non-concentrated listener. cf. Fig. 3 for an explanation of the abbreviations.

|  |  | FC | | | MC | | | NC | | |
| --- | --- | --- | --- | --- | --- | --- | --- | --- | --- | --- |
| Group I | Group II | p_min_ = 0 | p_min_ = 0.05 | p_min_ = 0.1 | p_min_ = 0 | p_min_ = 0.05 | p_min_ = 0.1 | p_min_ = 0 | p_min_ = 0.05 | p_min_ = 0.1 |
| SIUD | GRaBr | 23.8**** | 26.9**** | 25.6**** | 20.7**** | 19.7**** | 23.1**** | 19.2**** | 18.5**** | 18.2**** |
| SIUD | APTA | 17.4**** | 19.1**** | 21.4**** | 20.4**** | 22.3**** | 24.8**** | 22.0**** | 24.0**** | 26.7**** |
| SIUD | QUEST+ | 27.6**** | 28.9**** | 27.2**** | 23.5**** | 23.6**** | 25.5**** | 21.2**** | 21.0**** | 20.8**** |
| SIUD | UML | 27.6**** | 29.4**** | 27.5**** | 22.4**** | 22.1**** | 23.7**** | 15.7**** | 15.7**** | 17.1**** |
| SIUD | MLP | 3.5** | 2.8ns | 0.1ns | -2.2ns | -2.2ns | 0.0ns | -2.3ns | -1.5ns | 0.3ns |
| SIUD | SIAM | -32.5**** | -31.0**** | -32.3**** | -27.3**** | -27.3**** | -29.2**** | -37.0**** | -35.9**** | -35.3**** |
| GRaBr | APTA | 4.7**** | 7.3**** | 12.3**** | 5.8**** | 11.1**** | 14.8**** | 9.2**** | 14.4**** | 19.2**** |
| GRaBr | QUEST+ | 8.5**** | 6.3**** | 7.0**** | 7.2**** | 10.0**** | 7.2**** | 6.7**** | 8.0**** | 8.7**** |
| GRaBr | UML | 8.1**** | 6.1**** | 7.6**** | 5.0**** | 6.5**** | 5.2**** | **-2.6ns** | **-1.5ns** | **1.4ns** |
| GRaBr | MLP | -29.8**** | -34.0**** | -32.2**** | -16.8**** | -16.6**** | -17.5**** | -17.7**** | -17.1**** | -17.4**** |
| GRaBr | SIAM | -56.3**** | -55.3**** | -54.0**** | -38.8**** | -38.8**** | -41.9**** | -48.9**** | -48.1**** | -50.0**** |
| APTA | QUEST+ | -1.4ns | -5.1**** | -9.7**** | -2.5ns | -7.2**** | -12.2**** | -5.4**** | -10.9**** | -16.4**** |
| APTA | UML | -1.8ns | -5.4**** | -9.5**** | -3.1* | -8.2**** | -12.3**** | -8.8**** | -13.3**** | -17.5**** |
| APTA | MLP | -16.8**** | -18.7**** | -21.9**** | -17.9**** | -20.4**** | -22.8**** | -20.3**** | -22.7**** | -26.2**** |
| APTA | SIAM | -41.4**** | -38.6**** | -38.9**** | -38.8**** | -40.1**** | -42.4**** | -50.0**** | -50.4**** | -52.2**** |
| QUEST+ | UML | -0.8ns | -0.6ns | 0.7ns | -1.3ns | -2.4ns | -0.8ns | -6.0**** | -6.0**** | -4.0** |
| QUEST+ | MLP | -33.9**** | -34.8**** | -31.9**** | -18.7**** | -19.5**** | -19.9**** | -19.2**** | -19.2**** | -20.0**** |
| QUEST+ | SIAM | -58.5**** | -56.1**** | -54.0**** | -40.0**** | -40.7**** | -43.1**** | -49.8**** | -49.2**** | -51.3**** |
| UML | MLP | -34.3**** | -36.2**** | -32.1**** | -18.1**** | -18.5**** | -18.8**** | -15.3**** | -15.1**** | -16.4**** |
| UML | SIAM | -58.7**** | -56.7**** | -54.1**** | -39.6**** | -40.0**** | -42.3**** | -46.9**** | -46.3**** | -48.7**** |
| MLP | SIAM | -39.4**** | -36.5**** | -34.7**** | -23.6**** | -23.6**** | -27.5**** | -33.5**** | -33.5**** | -35.4**** |

^a^ ns: not significant; *p<0.05; **p<0.01; ***p<0.001; ****p<0.0001

TABLE S3. Statistical analysis of pair-wise t-test (t value and p value significance^a^) on the **root mean square error** of the threshold estimates L_50_ between different adaptive procedures simulated for the fully-, moderately-, and non-concentrated listener, for the **long-term inattentive listener**. The higher the t value is, the larger the difference between the adaptive procedure is. The ‘-’ sign in t value denotes a negative difference. FC: Fully-concentrated listener; MC: Moderately-concentrated listener; NC: Non-concentrated listener. cf. Fig. 3 for an explanation of the abbreviations.

|  |  | FC | | | MC | | | NC | | |
| --- | --- | --- | --- | --- | --- | --- | --- | --- | --- | --- |
| Group I | Group II | p_min_ = 0 | p_min_ = 0.05 | p_min_ = 0.1 | p_min_ = 0 | p_min_ = 0.05 | p_min_ = 0.1 | p_min_ = 0 | p_min_ = 0.05 | p_min_ = 0.1 |
| SIUD | GRaBr | 281.4**** | 238.8**** | 260.4**** | 173.9**** | 108.0**** | 121.1**** | 138.4**** | 158.0**** | 131.6**** |
| SIUD | APTA | -57.2**** | -85.6**** | -120.1**** | 18.8**** | -0.2ns | -44.3**** | 79.5**** | 60.0**** | 8.2**** |
| SIUD | QUEST+ | 252.0**** | 215.3**** | 86.5**** | 161.9**** | 99.9**** | 91.9**** | 123.8**** | 133.1**** | 101.8**** |
| SIUD | UML | 262.0**** | 215.2**** | 107.6**** | 93.6**** | 58.8**** | 57.7**** | 55.7**** | 42.7**** | 40.3**** |
| SIUD | MLP | 72.1**** | 72.7**** | 79.2**** | -50.6**** | -39.7**** | -46.5**** | -59.6**** | -69.6**** | -59.8**** |
| SIUD | SIAM | -309.0**** | -284.2**** | -297.2**** | -235.1**** | -178.6**** | -209.1**** | -311.6**** | -311.9**** | -316.7**** |
| GRaBr | APTA | -271.6**** | -228.9**** | -200.0**** | -282.4**** | -240.8**** | -203.3**** | -255.4**** | -200.4**** | -182.7**** |
| GRaBr | QUEST+ | -54.8**** | -45.0**** | -59.1**** | -55.7**** | -60.0**** | -54.6**** | -33.3**** | -39.5**** | -57.6**** |
| GRaBr | UML | -36.6**** | -31.5**** | -45.0**** | -33.6**** | -44.8**** | -58.2**** | -72.1**** | -93.7**** | -93.6**** |
| GRaBr | MLP | -246.8**** | -273.1**** | -281.7**** | -114.4**** | -111.3**** | -127.4**** | -175.1**** | -187.6**** | -185.2**** |
| GRaBr | SIAM | -577.2**** | -576.6**** | -577.3**** | -295.6**** | -244.3**** | -279.0**** | -440.6**** | -417.2**** | -432.5**** |
| APTA | QUEST+ | 250.9**** | 216.8**** | 155.7**** | 258.8**** | 220.4**** | 162.9**** | 132.9**** | 128.9**** | 129.4**** |
| APTA | UML | 257.9**** | 217.8**** | 165.5**** | 96.7**** | 84.1**** | 111.3**** | -7.4**** | -3.9** | 39.9**** |
| APTA | MLP | 117.9**** | 136.2**** | 148.3**** | -60.0**** | -44.6**** | -16.5**** | -131.7**** | -121.5**** | -75.1**** |
| APTA | SIAM | -216.1**** | -115.8**** | -9.2**** | -248.1**** | -191.9**** | -189.1**** | -404.6**** | -366.5**** | -344.4**** |
| QUEST+ | UML | 18.3**** | 8.5**** | 12.9**** | -22.9**** | -34.4**** | -27.4**** | -58.2**** | -73.6**** | -60.8**** |
| QUEST+ | MLP | -209.6**** | -227.7**** | -44.2**** | -110.3**** | -106.5**** | -110.7**** | -164.5**** | -171.9**** | -159.1**** |
| QUEST+ | SIAM | -554.2**** | -551.6**** | -307.4**** | -292.1**** | -240.6**** | -265.7**** | -429.3**** | -403.6**** | -411.4**** |
| UML | MLP | -222.2**** | -222.5**** | -65.3**** | -95.1**** | -82.5**** | -89.0**** | -107.6**** | -102.3**** | -99.1**** |
| UML | SIAM | -562.1**** | -546.5**** | -337.6**** | -271.7**** | -216.7**** | -244.8**** | -358.7**** | -335.6**** | -353.9**** |
| MLP | SIAM | -390.8**** | -407.1**** | -410.9**** | -153.5**** | -129.2**** | -154.2**** | -236.3**** | -233.0**** | -257.4**** |

^a^ ns: not significant; *p<0.05; **p<0.01; ***p<0.001; ****p<0.0001

TABLE S4. Statistical analysis of pair-wise t-test (t value and p value significance^a^) on the **root mean square error** of the threshold estimates L_50_ between different adaptive procedures simulated for the fully-, moderately-, and non-concentrated listener, for the **short-term inattentive listener**. The higher the t value is, the larger the difference between the adaptive procedure is. The ‘-’ sign in t value denotes a negative difference. FC: Fully-concentrated listener; MC: Moderately-concentrated listener; NC: Non-concentrated listener. cf. Fig. 3 for an explanation of the abbreviations.

|  |  | FC | | | MC | | | NC | | |
| --- | --- | --- | --- | --- | --- | --- | --- | --- | --- | --- |
| Group I | Group II | p_min_ = 0 | p_min_ = 0.05 | p_min_ = 0.1 | p_min_ = 0 | p_min_ = 0.05 | p_min_ = 0.1 | p_min_ = 0 | p_min_ = 0.05 | p_min_ = 0.1 |
| SIUD | GRaBr | 254.6**** | 271.2**** | 280.6**** | 124.9**** | 117.3**** | 140.2**** | 118.0**** | 132.9**** | 137.8**** |
| SIUD | APTA | -46.2**** | -87.8**** | -124.2**** | 38.3**** | -1.3ns | -62.3**** | 58.2**** | 8.2**** | -55.0**** |
| SIUD | QUEST+ | 222.1**** | 203.7**** | 105.0**** | 118.7**** | 105.1**** | 91.6**** | 106.1**** | 111.1**** | 102.5**** |
| SIUD | UML | 238.4**** | 238.5**** | 79.7**** | 100.7**** | 95.1**** | 70.8**** | 50.2**** | 57.9**** | 67.0**** |
| SIUD | MLP | 83.5**** | 74.7**** | 30.8**** | -40.2**** | -37.4**** | -25.9**** | -53.9**** | -45.1**** | -23.7**** |
| SIUD | SIAM | -294.5**** | -282.3**** | -326.1**** | -197.8**** | -182.1**** | -210.5**** | -300.5**** | -297.3**** | -281.8**** |
| GRaBr | APTA | -277.0**** | -228.0**** | -203.4**** | -240.4**** | -195.2**** | -144.9**** | -147.6**** | -140.7**** | -182.6**** |
| GRaBr | QUEST+ | -59.5**** | -75.5**** | -69.1**** | -38.7**** | -55.6**** | -68.2**** | -36.1**** | -58.6**** | -75.3**** |
| GRaBr | UML | -37.3**** | -62.6**** | -54.6**** | -31.1**** | -49.1**** | -73.7**** | -81.6**** | -95.1**** | -110.0**** |
| GRaBr | MLP | -275.7**** | -302.6**** | -129.8**** | -122.7**** | -125.1**** | -103.2**** | -184.0**** | -172.4**** | -167.4**** |
| GRaBr | SIAM | -593.0**** | -544.4**** | -554.9**** | -312.7**** | -297.2**** | -318.0**** | -481.8**** | -467.9**** | -500.0**** |
| APTA | QUEST+ | 249.4**** | 200.5**** | 164.8**** | 221.4**** | 172.7**** | 120.6**** | 106.6**** | 114.8**** | 152.9**** |
| APTA | UML | 263.2**** | 213.5**** | 154.6**** | 123.2**** | 147.4**** | 109.0**** | -1.1ns | 53.7**** | 121.4**** |
| APTA | MLP | 128.1**** | 132.9**** | 131.2**** | -70.0**** | -40.8**** | 28.9**** | -124.6**** | -55.2**** | 33.4**** |
| APTA | SIAM | -230.3**** | -107.8**** | -27.0**** | -253.8**** | -202.2**** | -115.2**** | -418.9**** | -318.4**** | -201.2**** |
| QUEST+ | UML | 25.6**** | 28.7**** | -2.8ns | -18.3**** | -16.3**** | -18.3**** | -65.1**** | -63.6**** | -49.4**** |
| QUEST+ | MLP | -214.7**** | -182.3**** | -54.7**** | -119.0**** | -117.0**** | -80.6**** | -171.7**** | -153.2**** | -131.5**** |
| QUEST+ | SIAM | -559.4**** | -481.4**** | -365.0**** | -308.8**** | -288.5**** | -285.8**** | -466.8**** | -446.3**** | -452.9**** |
| UML | MLP | -246.4**** | -244.9**** | -44.2**** | -109.9**** | -110.6**** | -70.2**** | -109.8**** | -103.1**** | -95.0**** |
| UML | SIAM | -577.8**** | -517.7**** | -304.7**** | -292.0**** | -279.2**** | -268.4**** | -380.1**** | -378.4**** | -401.2**** |
| MLP | SIAM | -423.2**** | -380.0**** | -283.3**** | -127.8**** | -124.6**** | -144.2**** | -244.1**** | -240.2**** | -256.4**** |

^a^ ns: not significant; *p<0.05; **p<0.01; ***p<0.001; ****p<0.0001

TABLE S5. Statistical analysis of pair-wise t-test (t value and p value significance^a^) on the **normalized efficienc**y of the threshold estimates L_50_ between different adaptive procedures simulated for the fully-, moderately-, and non-concentrated listener, for the **long-term inattentive listener**. The higher the t value is, the larger the difference between the adaptive procedure is. The ‘-’ sign in t value denotes a negative difference. FC: Fully-concentrated listener; MC: Moderately-concentrated listener; NC: Non-concentrated listener. cf. Fig. 3 for an explanation of the abbreviations.

|  |  | FC | | | MC | | | NC | | |
| --- | --- | --- | --- | --- | --- | --- | --- | --- | --- | --- |
| Group I | Group II | p_min_ = 0 | p_min_ = 0.05 | p_min_ = 0.1 | p_min_ = 0 | p_min_ = 0.05 | p_min_ = 0.1 | p_min_ = 0 | p_min_ = 0.05 | p_min_ = 0.1 |
| SIUD | GRaBr | -1.9**** | -2.0**** | -1.9**** | -2.2**** | -2.2**** | -2.3**** | -2.4**** | -2.3**** | -2.4**** |
| SIUD | APTA | 1.5**** | 1.7**** | 2.1**** | 0.4**** | 0.2**** | 1.1**** | -3.2**** | -1.4**** | -0.4**** |
| SIUD | QUEST+ | -10.2**** | -10.3**** | -4.0**** | -17.5**** | -21.1**** | -13.5**** | -25.6**** | -21.8**** | -17.6**** |
| SIUD | UML | -11.5**** | -11.1**** | -5.8**** | -8.7**** | -7.5**** | -6.0**** | -5.7**** | -2.5**** | -3.5**** |
| SIUD | MLP | -6.2**** | -6.8**** | -7.1**** | 1.6**** | 1.2**** | 1.3**** | 1.2**** | 1.3**** | 1.1**** |
| SIUD | SIAM | 0.6**** | 0.5**** | 0.7**** | 2.3**** | 2.2**** | 2.3**** | 2.1**** | 2.0**** | 2.1**** |
| GRaBr | APTA | 2.3**** | 2.5**** | 2.5**** | 2.3**** | 2.2**** | 2.5**** | 2.2**** | 2.2**** | 2.3**** |
| GRaBr | QUEST+ | -0.5**** | -1.0**** | 1.0**** | -0.4**** | -0.3**** | 0.8**** | 0.5**** | 0.8**** | 1.3**** |
| GRaBr | UML | -0.9**** | -1.3**** | 0.6**** | 1.1**** | 1.5**** | 1.7**** | 2.2**** | 2.2**** | 2.2**** |
| GRaBr | MLP | 0.6**** | 0.2**** | 0.3**** | 2.4**** | 2.3**** | 2.5**** | 2.5**** | 2.4**** | 2.4**** |
| GRaBr | SIAM | 2.1**** | 2.2**** | 2.1**** | 2.5**** | 2.4**** | 2.6**** | 2.5**** | 2.5**** | 2.5**** |
| APTA | QUEST+ | -27.4**** | -26.1**** | -23.5**** | -26.5**** | -24.5**** | -22.9**** | -21.1**** | -15.9**** | -16.4**** |
| APTA | UML | -28.0**** | -26.7**** | -25.8**** | -18.9**** | -10.4**** | -13.7**** | -0.6**** | 0.0** | -2.7**** |
| APTA | MLP | -45.5**** | -29.9**** | -34.5**** | 3.2**** | 1.3**** | 0.2**** | 5.1**** | 2.5**** | 1.7**** |
| APTA | SIAM | -0.7**** | -0.9**** | -1.1**** | 4.7**** | 2.8**** | 2.3**** | 5.6**** | 3.0**** | 2.6**** |
| QUEST+ | UML | -2.7**** | -1.7**** | -5.5**** | 15.0**** | 20.8**** | 14.8**** | 26.2**** | 27.0**** | 23.4**** |
| QUEST+ | MLP | 11.3**** | 9.3**** | -10.3**** | 30.7**** | 30.5**** | 29.6**** | 31.1**** | 30.9**** | 30.1**** |
| QUEST+ | SIAM | 10.8**** | 10.0**** | 5.0**** | 31.0**** | 30.8**** | 29.8**** | 31.1**** | 30.7**** | 29.0**** |
| UML | MLP | 13.6**** | 10.9**** | -3.5**** | 29.5**** | 27.4**** | 27.0**** | 26.9**** | 24.3**** | 24.0**** |
| UML | SIAM | 12.2**** | 10.7**** | 6.7**** | 28.7**** | 25.1**** | 25.4**** | 19.6**** | 14.7**** | 15.1**** |
| MLP | SIAM | 7.0**** | 6.8**** | 8.0**** | 4.4**** | 4.7**** | 5.4**** | 3.0**** | 3.0**** | 3.3**** |

^a^ ns: not significant; *p<0.05; **p<0.01; ***p<0.001; ****p<0.0001

TABLE S6. Statistical analysis of pair-wise t-test (t value and p value significance^a^) on the **normalized efficiency** of the threshold estimates L_50_ between different adaptive procedures simulated for the fully-, moderately-, and non-concentrated listener, for the **short-term inattentive listener**. The higher the t value is, the larger the difference between the adaptive procedure is. The ‘-’ sign in t value denotes a negative difference. FC: Fully-concentrated listener; MC: Moderately-concentrated listener; NC: Non-concentrated listener. cf. Fig. 3 for an explanation of the abbreviations.

|  |  | FC | | | MC | | | NC | | |
| --- | --- | --- | --- | --- | --- | --- | --- | --- | --- | --- |
| Group I | Group II | p_min_ = 0 | p_min_ = 0.05 | p_min_ = 0.1 | p_min_ = 0 | p_min_ = 0.05 | p_min_ = 0.1 | p_min_ = 0 | p_min_ = 0.05 | p_min_ = 0.1 |
| SIUD | GRaBr | -2.0**** | -2.0**** | -1.8**** | -1.9**** | -1.8**** | -1.8**** | -1.7**** | -1.6**** | -1.6**** |
| SIUD | APTA | 1.4**** | 1.9**** | 2.1**** | -0.6**** | 0.3**** | 1.5**** | -1.6**** | -0.2**** | 0.8**** |
| SIUD | QUEST+ | -11.4**** | -8.3**** | -4.0**** | -19.4**** | -16.3**** | -7.0**** | -19.3**** | -15.1**** | -12.3**** |
| SIUD | UML | -13.5**** | -11.0**** | -3.6**** | -13.9**** | -12.7**** | -4.2**** | -2.9**** | -3.1**** | -3.9**** |
| SIUD | MLP | -8.2**** | -7.2**** | -4.6**** | 1.1**** | 0.9**** | 0.8**** | 0.9**** | 0.7**** | 0.3**** |
| SIUD | SIAM | 0.5**** | 0.7**** | 0.8**** | 1.7**** | 1.6**** | 1.7**** | 1.5**** | 1.4**** | 1.2**** |
| GRaBr | APTA | 2.3**** | 2.4**** | 2.4**** | 1.8**** | 1.8**** | 2.0**** | 1.5**** | 1.6**** | 1.7**** |
| GRaBr | QUEST+ | -0.3**** | 0.1**** | 0.8**** | -0.5**** | 0.0* | 0.8**** | 0.2**** | 0.6**** | 1.0**** |
| GRaBr | UML | -0.8**** | -0.6**** | 0.9**** | 0.4**** | 0.5**** | 1.2**** | 1.5**** | 1.4**** | 1.4**** |
| GRaBr | MLP | 0.5**** | 0.5**** | 0.6**** | 2.0**** | 1.9**** | 1.9**** | 1.7**** | 1.7**** | 1.6**** |
| GRaBr | SIAM | 2.1**** | 2.2**** | 2.1**** | 2.1**** | 2.0**** | 2.0**** | 1.8**** | 1.7**** | 1.7**** |
| APTA | QUEST+ | -27.0**** | -25.0**** | -23.9**** | -20.3**** | -22.9**** | -23.4**** | -13.8**** | -19.2**** | -23.2**** |
| APTA | UML | -28.0**** | -27.1**** | -23.3**** | -14.7**** | -19.4**** | -18.7**** | -0.1**** | -4.2**** | -11.3**** |
| APTA | MLP | -46.2**** | -33.4**** | -30.3**** | 2.2**** | 1.1**** | -3.0**** | 2.7**** | 1.4**** | -1.6**** |
| APTA | SIAM | -0.6**** | -0.9**** | -1.1**** | 2.8**** | 2.1**** | 0.7**** | 3.0**** | 2.2**** | 0.7**** |
| QUEST+ | UML | -3.9**** | -5.8**** | 1.2**** | 9.4**** | 6.4**** | 8.6**** | 25.9**** | 23.1**** | 18.6**** |
| QUEST+ | MLP | 9.3**** | 3.9**** | -2.2**** | 30.6**** | 30.2**** | 26.9**** | 30.8**** | 30.1**** | 28.5**** |
| QUEST+ | SIAM | 10.5**** | 9.0**** | 5.5**** | 29.3**** | 27.9**** | 20.2**** | 29.2**** | 26.6**** | 20.5**** |
| UML | MLP | 12.8**** | 10.1**** | -3.6**** | 29.9**** | 29.6**** | 24.3**** | 25.3**** | 24.1**** | 22.2**** |
| UML | SIAM | 12.4**** | 11.7**** | 5.2**** | 26.7**** | 25.6**** | 15.2**** | 11.8**** | 11.2**** | 9.3**** |
| MLP | SIAM | 7.5**** | 8.0**** | 6.2**** | 2.1**** | 2.2**** | 2.9**** | 1.8**** | 1.9**** | 2.0**** |

^a^ ns: not significant; *p<0.05; **p<0.01; ***p<0.001; ****p<0.0001

TABLE S7. Statistical analysis of pair-wise t-test (t value and p value significance^a^) on the **rate of convergence** of the threshold estimates L_50_ between different adaptive procedures simulated for the fully-, moderately-, and non-concentrated listener, for the **long-term inattentive listener**. The higher the t value is, the larger the difference between the adaptive procedure is. The ‘-’ sign in t value denotes a negative difference. FC: Fully-concentrated listener; MC: Moderately-concentrated listener; NC: Non-concentrated listener. cf. Fig. 3 for an explanation of the abbreviations.

|  |  | FC | | | MC | | | NC | | |
| --- | --- | --- | --- | --- | --- | --- | --- | --- | --- | --- |
| Group I | Group II | p_min_ = 0 | p_min_ = 0.05 | p_min_ = 0.1 | p_min_ = 0 | p_min_ = 0.05 | p_min_ = 0.1 | p_min_ = 0 | p_min_ = 0.05 | p_min_ = 0.1 |
| SIUD | GRaBr | 3.7* | 3.4* | 3.4* | 5.2*** | 4.7*** | 4.7** | 5.7**** | 5.4**** | 5.5**** |
| SIUD | APTA | -4.1* | -5.2*** | -6.5**** | 2.4ns | -0.2ns | -2.4ns | 5.0** | 2.6ns | -0.1ns |
| SIUD | QUEST+ | 3.9* | 2.7ns | 1.6ns | 5.3*** | 4.6** | 4.0* | 6.3**** | 5.2*** | 4.6** |
| SIUD | UML | 4.1* | 2.9ns | 1.4ns | 2.5ns | 2.4ns | 1.8ns | 1.6ns | 1.4ns | 1.1ns |
| SIUD | MLP | 2.5ns | 2.0ns | 1.2ns | -2.0ns | -2.2ns | -1.9ns | -4.0* | -4.1** | -3.8* |
| SIUD | SIAM | -1.5ns | -1.7ns | -2.0ns | -8.5**** | -8.6**** | -8.3**** | -17.1**** | -16.9**** | -16.5**** |
| GRaBr | APTA | -8.4**** | -7.7**** | -8.3**** | -5.9*** | -6.3**** | -7.3**** | **-2.8ns** | -4.2** | -6.2**** |
| GRaBr | QUEST+ | -0.2ns | -0.5ns | -1.2ns | -0.1ns | -0.2ns | -0.9ns | 0.0ns | -0.2ns | -1.0ns |
| GRaBr | UML | 0.2ns | -0.2ns | -1.2ns | -1.7ns | -1.6ns | -1.8ns | -3.5ns | -3.0ns | -3.5ns |
| GRaBr | MLP | -0.8ns | -0.8ns | -0.9ns | -7.3*** | -7.4**** | -6.9*** | -11.3**** | -11.0**** | -10.9**** |
| GRaBr | SIAM | -4.3** | -4.3** | -4.6** | -15.6**** | -15.5**** | -15.5**** | -30.2**** | -30.8**** | -32.2**** |
| APTA | QUEST+ | 9.9*** | 7.0**** | 7.1**** | 6.6** | 6.2**** | 6.6**** | 4.1ns | 4.0* | 5.2*** |
| APTA | UML | 9.8*** | 7.0**** | 6.8**** | 1.3ns | 2.9ns | 3.8* | -2.3ns | -0.5ns | 1.2ns |
| APTA | MLP | 6.2** | 6.0*** | 6.2**** | -5.0* | -2.5ns | 0.5ns | -13.4**** | -8.7**** | -4.1* |
| APTA | SIAM | 0.9ns | 2.8ns | 4.6** | -14.7**** | -10.1**** | -5.6**** | -83.3**** | -37.7**** | -19.3**** |
| QUEST+ | UML | 0.4ns | 0.3ns | -0.1ns | -1.7ns | -1.4ns | -1.3ns | -3.8ns | -2.9ns | -2.7ns |
| QUEST+ | MLP | -0.8ns | -0.4ns | 0.0ns | -7.5*** | -7.4*** | -6.2** | -13.2**** | -10.8**** | -9.7**** |
| QUEST+ | SIAM | -4.4** | -3.7* | -3.1ns | -16.1**** | -15.7**** | -14.6**** | -41.4**** | -30.8**** | -29.2**** |
| UML | MLP | -1.1ns | -0.6ns | 0.1ns | -4.3* | -4.5* | -3.4ns | -5.4** | -5.0** | -4.4* |
| UML | SIAM | -4.6** | -3.8* | -2.8ns | -10.3**** | -10.4**** | -9.0**** | -17.1**** | -15.4**** | -14.3**** |
| MLP | SIAM | -3.3ns | -3.1ns | -2.5ns | -6.2*** | -6.2*** | -6.2*** | -16.0**** | -15.0**** | -14.8**** |

^a^ ns: not significant; *p<0.05; **p<0.01; ***p<0.001; ****p<0.0001

TABLE S8. Statistical analysis of pair-wise t-test (t value and p value significance^a^) on the **rate of convergence** of the threshold estimates L_50_ between different adaptive procedures simulated for the fully-, moderately-, and non-concentrated listener, for the **short-term inattentive listener**. The higher the t value is, the larger the difference between the adaptive procedure is. The ‘-’ sign in t value denotes a negative difference. FC: Fully-concentrated listener; MC: Moderately-concentrated listener; NC: Non-concentrated listener. cf. Fig. 3 for an explanation of the abbreviations.

|  |  | FC | | | MC | | | NC | | |
| --- | --- | --- | --- | --- | --- | --- | --- | --- | --- | --- |
| Group I | Group II | p_min_ = 0 | p_min_ = 0.05 | p_min_ = 0.1 | p_min_ = 0 | p_min_ = 0.05 | p_min_ = 0.1 | p_min_ = 0 | p_min_ = 0.05 | p_min_ = 0.1 |
| SIUD | GRaBr | 3.5* | 3.4* | 3.1ns | 5.3*** | 5.3*** | 5.2*** | 5.1*** | 4.8*** | 4.8*** |
| SIUD | APTA | -4.3** | -5.1*** | -6.7**** | 1.5ns | -1.4ns | -5.0*** | 3.0ns | -0.5ns | -5.7**** |
| SIUD | QUEST+ | 4.1** | 3.2ns | 1.5ns | 5.1*** | 4.6** | 2.9ns | 6.6**** | 5.6*** | 4.5** |
| SIUD | UML | 3.8* | 2.8ns | 1.6ns | 3.0ns | 2.6ns | 1.6ns | 2.5ns | 2.3ns | 1.3ns |
| SIUD | MLP | 2.7ns | 2.3ns | 1.2ns | -1.7ns | -1.7ns | -1.8ns | -2.7ns | -2.7ns | -2.9ns |
| SIUD | SIAM | -1.5ns | -1.6ns | -2.1ns | -5.6**** | -5.6**** | -5.8**** | -13.2**** | -12.4**** | -13.1**** |
| GRaBr | APTA | -8.1**** | -7.6**** | -8.4**** | -6.3**** | -6.5**** | -10.2**** | **-3.0ns** | -5.5**** | -10.9**** |
| GRaBr | QUEST+ | 0.0ns | -0.4ns | -1.0ns | -0.1ns | -0.5ns | -1.8ns | 0.4ns | -0.2ns | -1.1ns |
| GRaBr | UML | 0.1ns | -0.2ns | -0.9ns | -1.2ns | -1.3ns | -2.0ns | -2.1ns | -2.2ns | -2.8ns |
| GRaBr | MLP | -0.7ns | -0.8ns | -0.9ns | -6.9*** | -6.7*** | -5.8** | -8.2**** | -8.5**** | -7.7**** |
| GRaBr | SIAM | -4.2** | -4.2** | -4.5** | -12.2**** | -12.0**** | -11.6**** | -20.1**** | -18.6**** | -18.8**** |
| APTA | QUEST+ | 10.6*** | 7.6**** | 7.1**** | 6.0*** | 5.8*** | 7.8**** | 4.5** | 6.4**** | 12.2**** |
| APTA | UML | 9.0*** | 6.8**** | 7.1**** | 2.4ns | 3.7* | 5.8*** | 0.2ns | 2.8ns | 6.2** |
| APTA | MLP | 7.0** | 6.4**** | 6.5**** | -3.6ns | -0.3ns | 2.6ns | -6.6*** | -2.2ns | 2.4ns |
| APTA | SIAM | 1.0ns | 2.7ns | 4.8*** | -8.7**** | -3.9** | -0.5ns | -23.9**** | -12.4**** | -8.8**** |
| QUEST+ | UML | 0.1ns | 0.1ns | 0.1ns | -1.1ns | -0.9ns | -0.7ns | -2.8ns | -2.4ns | -2.2ns |
| QUEST+ | MLP | -0.8ns | -0.5ns | -0.1ns | -6.8*** | -6.0** | -4.2* | -10.8**** | -11.0**** | -7.9**** |
| QUEST+ | SIAM | -4.6** | -4.0** | -3.0ns | -11.9**** | -10.8**** | -8.7**** | -32.7**** | -27.8**** | -24.7**** |
| UML | MLP | -0.8ns | -0.5ns | -0.1ns | -4.5* | -3.9* | -2.9ns | -5.0** | -5.1** | -3.9* |
| UML | SIAM | -4.4** | -3.7* | -3.0ns | -8.3**** | -7.5**** | -6.4*** | -14.0**** | -13.6**** | -11.9**** |
| MLP | SIAM | -3.6* | -3.3ns | -2.5ns | -3.7* | -3.5ns | -3.1ns | -11.5**** | -14.6**** | -9.5*** |

^a^ ns: not significant; *p<0.05; **p<0.01; ***p<0.001; ****p<0.0001

TABLE S9. Statistical analysis of pair-wise t-test (t value and p value significance^a^) on the **absolute erro**r of the threshold estimates L_50_ between different adaptive procedures simulated for the fully-, moderately-, and non-concentrated listener, for the **long-term inattentive listener**. The higher the t value is, the larger the difference between the adaptive procedure is. The ‘-’ sign in t value denotes a negative difference. FC: Fully-concentrated listener; MC: Moderately-concentrated listener; NC: Non-concentrated listener. cf. Fig. 3 for an explanation of the abbreviations.

|  |  | FC | | | MC | | | NC | | |
| --- | --- | --- | --- | --- | --- | --- | --- | --- | --- | --- |
| Group I | Group II | p_min_ = 0 | p_min_ = 0.05 | p_min_ = 0.1 | p_min_ = 0 | p_min_ = 0.05 | p_min_ = 0.1 | p_min_ = 0 | p_min_ = 0.05 | p_min_ = 0.1 |
| SIUD | GRaBr | 26.2**** | 25.8**** | 28.4**** | 25.6**** | 21.3**** | 22.2**** | 20.7**** | 21.8**** | 19.7**** |
| SIUD | APTA | -7.1**** | -9.7**** | -13.4**** | 0.8ns | -2.7ns | -7.5**** | 8.8**** | 5.6**** | -0.6ns |
| SIUD | QUEST+ | 25.1**** | 23.3**** | 17.7**** | 23.5**** | 20.4**** | 17.8**** | 19.1**** | 19.5**** | 16.8**** |
| SIUD | UML | 24.0**** | 24.0**** | 21.1**** | 21.1**** | 15.4**** | 14.8**** | 13.6**** | 11.2**** | 10.0**** |
| SIUD | MLP | 4.9**** | 5.9**** | 7.2**** | -2.3ns | -2.6ns | -2.7ns | -4.3*** | -4.7**** | -4.5*** |
| SIUD | SIAM | -33.8**** | -33.4**** | -33.0**** | -28.5**** | -25.6**** | -26.8**** | -36.0**** | -35.8**** | -38.0**** |
| GRaBr | APTA | -31.8**** | -28.2**** | -25.5**** | -30.7**** | -29.4**** | -26.6**** | -27.4**** | -27.7**** | -24.0**** |
| GRaBr | QUEST+ | 0.3ns | -4.4*** | -11.2**** | -5.5**** | -1.4ns | -8.6**** | -5.6**** | -6.6**** | -5.6**** |
| GRaBr | UML | -4.5*** | -3.0ns | -6.8**** | -4.3*** | -6.7**** | -8.4**** | -6.7**** | -8.9**** | -9.5**** |
| GRaBr | MLP | -28.9**** | -26.0**** | -27.1**** | -14.4**** | -15.0**** | -15.1**** | -16.6**** | -17.2**** | -16.9**** |
| GRaBr | SIAM | -56.9**** | -56.9**** | -55.6**** | -35.0**** | -32.4**** | -33.2**** | -43.3**** | -43.1**** | -44.9**** |
| APTA | QUEST+ | 30.7**** | 26.6**** | 21.8**** | 28.0**** | 28.0**** | 23.2**** | 22.5**** | 22.8**** | 20.3**** |
| APTA | UML | 29.9**** | 27.1**** | 23.2**** | 24.1**** | 21.0**** | 20.6**** | 8.9**** | 8.2**** | 11.6**** |
| APTA | MLP | 12.4**** | 14.7**** | 17.1**** | -2.9ns | -0.9ns | 2.9ns | -10.0**** | -8.4**** | -4.3*** |
| APTA | SIAM | -26.5**** | -19.6**** | -8.4**** | -29.0**** | -25.0**** | -23.3**** | -39.8**** | -38.4**** | -38.2**** |
| QUEST+ | UML | -4.0** | 1.3ns | 3.8** | -0.9ns | -5.5**** | -2.5ns | -4.6*** | -6.4**** | -6.4**** |
| QUEST+ | MLP | -26.4**** | -22.3**** | -12.8**** | -13.5**** | -14.7**** | -13.0**** | -15.8**** | -16.1**** | -15.4**** |
| QUEST+ | SIAM | -55.7**** | -54.9**** | -47.6**** | -34.6**** | -32.2**** | -32.2**** | -42.9**** | -42.5**** | -44.1**** |
| UML | MLP | -25.7**** | -23.4**** | -17.0**** | -13.1**** | -12.3**** | -11.8**** | -13.2**** | -12.0**** | -11.5**** |
| UML | SIAM | -55.4**** | -55.5**** | -50.1**** | -34.3**** | -31.0**** | -31.5**** | -41.3**** | -40.0**** | -42.0**** |
| MLP | SIAM | -40.4**** | -40.7**** | -40.3**** | -25.1**** | -22.6**** | -23.7**** | -31.2**** | -30.8**** | -33.4**** |

^a^ ns: not significant; *p<0.05; **p<0.01; ***p<0.001; ****p<0.0001

TABLE S10. Statistical analysis of pair-wise t-test (t value and p value significance^a^) on the **absolute erro**r of the threshold estimates L_50_ between different adaptive procedures simulated for the fully-, moderately-, and non-concentrated listener, for the **short-term inattentive listener**. The higher the t value is, the larger the difference between the adaptive procedure is. The ‘-’ sign in t value denotes a negative difference. FC: Fully-concentrated listener; MC: Moderately-concentrated listener; NC: Non-concentrated listener. cf. Fig. 3 for an explanation of the abbreviations.

|  |  | FC | | | MC | | | NC | | |
| --- | --- | --- | --- | --- | --- | --- | --- | --- | --- | --- |
| Group I | Group II | p_min_ = 0 | p_min_ = 0.05 | p_min_ = 0.1 | p_min_ = 0 | p_min_ = 0.05 | p_min_ = 0.1 | p_min_ = 0 | p_min_ = 0.05 | p_min_ = 0.1 |
| SIUD | GRaBr | 26.8**** | 28.3**** | 28.4**** | 20.5**** | 20.0**** | 21.5**** | 18.0**** | 17.4**** | 17.0**** |
| SIUD | APTA | -5.6**** | -9.4**** | -13.9**** | 4.3*** | -1.5ns | -8.4**** | 8.6**** | 2.1ns | -5.4**** |
| SIUD | QUEST+ | 25.4**** | 22.8**** | 17.6**** | 20.4**** | 17.9**** | 16.1**** | 17.5**** | 15.3**** | 13.7**** |
| SIUD | UML | 24.8**** | 24.9**** | 18.6**** | 18.7**** | 17.4**** | 14.9**** | 12.1**** | 11.2**** | 10.7**** |
| SIUD | MLP | 6.6**** | 5.9**** | 5.0**** | -1.6ns | -1.4ns | 0.7ns | -2.4ns | -1.4ns | 0.3ns |
| SIUD | SIAM | -35.2**** | -32.1**** | -34.6**** | -27.2**** | -26.9**** | -29.3**** | -37.0**** | -36.0**** | -35.3**** |
| GRaBr | APTA | -30.9**** | -28.5**** | -26.3**** | -27.6**** | -25.9**** | -22.9**** | -22.0**** | -20.3**** | -20.6**** |
| GRaBr | QUEST+ | 0.0ns | -8.7**** | -11.3**** | 0.2ns | -6.2**** | -9.4**** | -1.7ns | -7.0**** | -9.8**** |
| GRaBr | UML | -4.2*** | -6.2**** | -7.1**** | -4.0** | -6.0**** | -8.3**** | -8.1**** | -9.5**** | -10.9**** |
| GRaBr | MLP | -28.8**** | -29.3**** | -24.2**** | -15.2**** | -14.8**** | -14.3**** | -16.6**** | -15.9**** | -16.1**** |
| GRaBr | SIAM | -60.9**** | -55.2**** | -55.1**** | -37.9**** | -37.7**** | -40.4**** | -48.2**** | -47.4**** | -49.0**** |
| APTA | QUEST+ | 29.6**** | 25.3**** | 22.4**** | 27.1**** | 23.3**** | 19.8**** | 20.1**** | 17.4**** | 17.9**** |
| APTA | UML | 29.1**** | 26.5**** | 23.0**** | 23.5**** | 22.5**** | 19.1**** | 7.0**** | 11.3**** | 15.4**** |
| APTA | MLP | 12.5**** | 14.2**** | 16.5**** | -4.8**** | -0.3ns | 8.0**** | -9.5**** | -3.4* | 5.6**** |
| APTA | SIAM | -29.0**** | -18.5**** | -10.5**** | -30.7**** | -26.8**** | -20.8**** | -43.8**** | -38.6**** | -29.5**** |
| QUEST+ | UML | -3.3* | 2.9ns | 2.3ns | -3.9** | -0.6ns | -0.7ns | -7.2**** | -5.7**** | -4.4*** |
| QUEST+ | MLP | -25.8**** | -21.3**** | -12.9**** | -15.2**** | -13.5**** | -10.9**** | -16.2**** | -14.3**** | -12.9**** |
| QUEST+ | SIAM | -59.2**** | -51.3**** | -48.0**** | -37.8**** | -36.8**** | -38.2**** | -48.0**** | -46.4**** | -47.1**** |
| UML | MLP | -25.6**** | -24.4**** | -14.2**** | -14.2**** | -13.2**** | -10.2**** | -12.4**** | -11.0**** | -10.1**** |
| UML | SIAM | -59.3**** | -52.9**** | -48.1**** | -37.1**** | -36.6**** | -37.6**** | -45.2**** | -44.0**** | -44.9**** |
| MLP | SIAM | -44.2**** | -38.8**** | -38.7**** | -23.7**** | -23.7**** | -27.8**** | -33.5**** | -33.5**** | -35.2**** |

^a^ ns: not significant; *p<0.05; **p<0.01; ***p<0.001; ****p<0.0001
