# Supplementary material for: How Does Inattention Influence the Robustness and Efficiency of Adaptive Procedures in the Context of Psychoacoustic Assessments via Smartphone?
Source: Trends Hear. 2024 Nov 18;28:23312165241288051. doi: 10.1177/23312165241288051 (PMC11574912; doi:10.1177/23312165241288051)
Supplement: sj-docx-2-tia-10.1177_23312165241288051 - Supplemental material for How Does Inattention Influence the Robustness and Efficiency of Adaptive Procedures in the Context of Psychoacoustic Assessments via Smartphone? [file sj-docx-2-tia-10.1177_23312165241288051.docx]

|  | $Absolute Error(k) =\left\vert\hat{L_{50,k}}- L_{50} \right\vert$ | (S1) |
| --- | --- | --- |

**Absolute error**: The absolute error (i.e., absolute difference) between the estimated threshold $\hat{L_{50}}$ and the true threshold L_50_ in the k_th_ simulation, which is considered as a measure of accuracy (Rinderknecht et al., 2018), is calculated using Eq. S1:


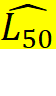


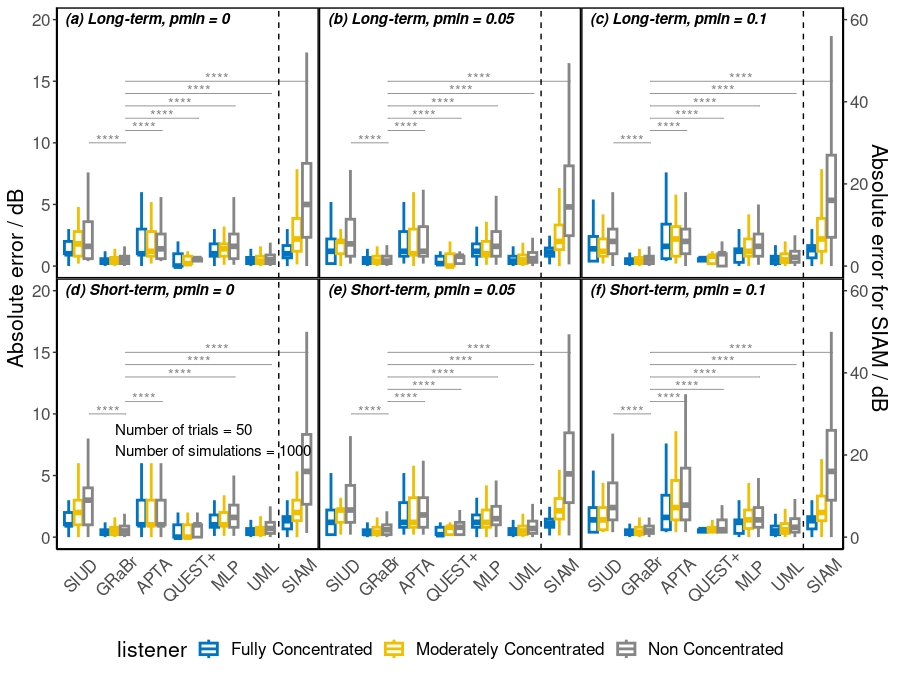


Fig. S1. Absolute error of seven adaptive procedures grouped by three levels of inattention for two types of inattentive listeners with three levels of false alarm rate. Statistical results of the pair-wise comparison against **GRaBr** for **the NC listener** are visualized. See Fig. 4 for an illustration of the box-plot and statistical labels and refer to Fig. 3 for an explanation of the abbreviations for the adaptive procedure. Note that the absolute error of SIAM is plotted with a different scale (given at the right side of the figure) since a scaling factor of 1/3 had to be applied to display the data into the same plot as other procedures.

Comparisons across seven adaptive procedures in terms of absolute error are presented in Fig. S1. The larger the absolute error is, the less robust the procedure is. It is evident that APTA, SIUD, MLP, SIAM produced a considerably large absolute error. Furthermore, absolute errors of GRaBr, QUEST+, and UML were small. Generally, GRaBr had a smaller absolute error than the baseline SIUD method. A similar trend was also observed between MLP and UML, where UML outperformed the baseline method MLP by producing smaller absolute errors. Similar to the bias, the performance for most adaptive procedures became worse as the level of inattention or false alarm rate increased.

All four factors (i.e., type of inattention, degree of inattention, false alarm rate, and adaptive procedure) were significantly influential on the absolute error, implied by a four-way ANOVA test (p < 0.05). Later, we performed a pair-wise t-test to compare different adaptive procedures. Results showed that most adaptive procedures significantly differed from each other in terms of the absolute error (p < 0.05).

However, there was no significant difference between GRaBr and QUEST+ for the long-term FC listener if p_min_ equaled to 0, for the long-term MC listener in case p_min_ was 0.05, and for the short-term listener if p_min_ was 0. GRaBr did not differ from UML for the long-term FC listener if p_min_ was 0.05. See the supplementary material (Tables S9 & S10) for the complete statistical comparison.
