# Supplementary material for: How Does Inattention Influence the Robustness and Efficiency of Adaptive Procedures in the Context of Psychoacoustic Assessments via Smartphone?
Source: Trends Hear. 2024 Nov 18;28:23312165241288051. doi: 10.1177/23312165241288051 (PMC11574912; doi:10.1177/23312165241288051)
Supplement: sj-docx-3-tia-10.1177_23312165241288051 - Supplemental material for How Does Inattention Influence the Robustness and Efficiency of Adaptive Procedures in the Context of Psychoacoustic Assessments via Smartphone? [file sj-docx-3-tia-10.1177_23312165241288051.docx]

TABLE S11. Results of the four-way analysis of variance (ANOVA) on the dependent variable **bias**. Four factors include type and degree of inattention, false alarm rate p_min_, and adaptive method. DFn and DFd stand for degrees of freedom in the numerator and denominator. F and p are F-value and p-value. Ges indicates generalized Eta-Squared measure of effect size.

| Effect | DFn | DFd | F | p | p<.05 | ges |
| --- | --- | --- | --- | --- | --- | --- |
| type | 1 | 121552 | 31 | 0.00 | * | 0.00 |
| degree | 2 | 121552 | 5049.9 | 0.00 | * | 0.08 |
| method | 6 | 121552 | 15549.2 | 0.00 | * | 0.43 |
| p_min_ | 2 | 121552 | 70.7 | 0.00 | * | 0.00 |
| type:degree | 2 | 121552 | 11.4 | 0.00 | * | 0.00 |
| type:method | 6 | 121552 | 19.4 | 0.00 | * | 0.00 |
| degree:method | 12 | 121552 | 2685.4 | 0.00 | * | 0.21 |
| type:p_min_ | 2 | 121552 | 9.9 | 0.00 | * | 0.00 |
| degree:p_min_ | 4 | 121552 | 2.1 | 0.08 |  | 0.00 |
| method:p_min_ | 12 | 121552 | 57 | 0.00 | * | 0.01 |
| type:degree:method | 12 | 121552 | 19 | 0.00 | * | 0.00 |
| type:degree:p_min_ | 4 | 121552 | 5.1 | 0.00 | * | 0.00 |
| type:method:p_min_ | 12 | 121552 | 3.5 | 0.00 | * | 0.00 |
| degree:method:p_min_ | 24 | 121552 | 3.9 | 0.00 | * | 0.00 |
| type:degree:method:p_min_ | 24 | 121552 | 3.4 | 0.00 | * | 0.00 |

TABLE S12. Results of the four-way analysis of variance (ANOVA) on the dependent variable **root-mean-square error (RMSE)**. Four factors include type and degree of inattention, false alarm rate p_min_, and adaptive method. DFn and DFd stand for degrees of freedom in the numerator and denominator. F and p are F-value and p-value. Ges indicates generalized Eta-Squared measure of effect size.

| Effect | DFn | DFd | F | p | p<.05 | ges |
| --- | --- | --- | --- | --- | --- | --- |
| type | 1 | 125874 | 1416.5 | 0.00 | * | 0.01 |
| degree | 2 | 125874 | 501377.8 | 0.00 | * | 0.89 |
| method | 6 | 125874 | 666776.2 | 0.00 | * | 0.97 |
| p_min_ | 2 | 125874 | 9494.0 | 0.00 | * | 0.13 |
| type:degree | 2 | 125874 | 3006.0 | 0.00 | * | 0.05 |
| type:method | 6 | 125874 | 3172.1 | 0.00 | * | 0.13 |
| degree:method | 12 | 125874 | 135663.3 | 0.00 | * | 0.93 |
| type:p_min_ | 2 | 125874 | 555.0 | 0.00 | * | 0.01 |
| degree:p_min_ | 4 | 125874 | 515.7 | 0.00 | * | 0.02 |
| method:p_min_ | 12 | 125874 | 3419.4 | 0.00 | * | 0.25 |
| type:degree:method | 12 | 125874 | 1380.0 | 0.00 | * | 0.12 |
| type:degree:p_min_ | 4 | 125874 | 598.7 | 0.00 | * | 0.02 |
| type:method:p_min_ | 12 | 125874 | 557.2 | 0.00 | * | 0.05 |
| degree:method:p_min_ | 24 | 125874 | 316.4 | 0.00 | * | 0.06 |
| type:degree:method:p_min_ | 24 | 125874 | 315.9 | 0.00 | * | 0.06 |

TABLE S13. Results of the four-way analysis of variance (ANOVA) on the dependent variable **normalized efficiency**. Four factors include type and degree of inattention, false alarm rate p_min_, and adaptive method. DFn and DFd stand for degrees of freedom in the numerator and denominator. F and p are F-value and p-value. Ges indicates generalized Eta-Squared measure of effect size.

| Effect | DFn | DFd | F | p | p<.05 | ges |
| --- | --- | --- | --- | --- | --- | --- |
| type | 1 | 121552 | 30053.9 | 0.00 | * | 0.20 |
| degree | 2 | 121552 | 1637871.9 | 0.00 | * | 0.96 |
| method | 6 | 121552 | 1112821.3 | 0.00 | * | 0.98 |
| p_min_ | 2 | 121552 | 246146.2 | 0.00 | * | 0.80 |
| type:degree | 2 | 121552 | 3178.2 | 0.00 | * | 0.05 |
| type:method | 6 | 121552 | 11377.5 | 0.00 | * | 0.36 |
| degree:method | 12 | 121552 | 179389.2 | 0.00 | * | 0.95 |
| type:p_min_ | 2 | 121552 | 1512.2 | 0.00 | * | 0.02 |
| degree:p_min_ | 4 | 121552 | 37013.0 | 0.00 | * | 0.55 |
| method:p_min_ | 12 | 121552 | 47787.9 | 0.00 | * | 0.83 |
| type:degree:method | 12 | 121552 | 4626.6 | 0.00 | * | 0.31 |
| type:degree:p_min_ | 4 | 121552 | 925.6 | 0.00 | * | 0.03 |
| type:method:p_min_ | 12 | 121552 | 2056.1 | 0.00 | * | 0.17 |
| degree:method:p_min_ | 24 | 121552 | 10213.9 | 0.00 | * | 0.67 |
| type:degree:method:p_min_ | 24 | 121552 | 943.9 | 0.00 | * | 0.16 |

TABLE S14. Results of the four-way analysis of variance (ANOVA) on the dependent variable **rate of convergence**. Four factors include type and degree of inattention, false alarm rate p_min_, and adaptive method. DFn and DFd stand for degrees of freedom in the numerator and denominator. F and p are F-value and p-value. Ges indicates generalized Eta-Squared measure of effect size.

| Effect | DFn | DFd | F | p | p<.05 | ges |
| --- | --- | --- | --- | --- | --- | --- |
| type | 1 | 1710 | 6.4 | 0.01 | * | 0.00 |
| degree | 2 | 1710 | 1168.4 | 0.00 | * | 0.58 |
| method | 6 | 1710 | 718.5 | 0.00 | * | 0.72 |
| p_min_ | 2 | 1710 | 57.9 | 0.00 | * | 0.06 |
| type:degree | 2 | 1710 | 11.3 | 0.00 | * | 0.01 |
| type:method | 6 | 1710 | 15.1 | 0.00 | * | 0.05 |
| degree:method | 12 | 1710 | 165.7 | 0.00 | * | 0.54 |
| type:p_min_ | 2 | 1710 | 2.1 | 0.12 |  | 0.00 |
| degree:p_min_ | 4 | 1710 | 0.7 | 0.56 |  | 0.00 |
| method:p_min_ | 12 | 1710 | 18.2 | 0.00 | * | 0.11 |
| type:degree:method | 12 | 1710 | 4.7 | 0.00 | * | 0.03 |
| type:degree:p_min_ | 4 | 1710 | 0.8 | 0.55 |  | 0.00 |
| type:method:p_min_ | 12 | 1710 | 1.0 | 0.46 |  | 0.01 |
| degree:method:p_min_ | 24 | 1710 | 0.4 | 1.00 |  | 0.01 |
| type:degree:method:p_min_ | 24 | 1710 | 0.4 | 1.00 |  | 0.01 |

TABLE S15. Results of the four-way analysis of variance (ANOVA) on the dependent variable **absolute error**. Four factors include type and degree of inattention, false alarm rate p_min_, and adaptive method. DFn and DFd stand for degrees of freedom in the numerator and denominator. F and p are F-value and p-value. Ges indicates generalized Eta-Squared measure of effect size.

| Effect | DFn | DFd | F | p | p<.05 | ges |
| --- | --- | --- | --- | --- | --- | --- |
| type | 1 | 121552 | 28.5 | 0.00 | * | 0.00 |
| degree | 2 | 121552 | 6660.4 | 0.00 | * | 0.10 |
| method | 6 | 121552 | 12987.8 | 0.00 | * | 0.39 |
| p_min_ | 2 | 121552 | 102.0 | 0.00 | * | 0.00 |
| type:degree | 2 | 121552 | 37.5 | 0.00 | * | 0.00 |
| type:method | 6 | 121552 | 17.9 | 0.00 | * | 0.00 |
| degree:method | 12 | 121552 | 2674.9 | 0.00 | * | 0.21 |
| type:p_min_ | 2 | 121552 | 4.9 | 0.01 | * | 0.00 |
| degree:p_min_ | 4 | 121552 | 4.9 | 0.00 | * | 0.00 |
| method:p_min_ | 12 | 121552 | 31.0 | 0.00 | * | 0.00 |
| type:degree:method | 12 | 121552 | 10.5 | 0.00 | * | 0.00 |
| type:degree:p_min_ | 4 | 121552 | 4.7 | 0.00 | * | 0.00 |
| type:method:p_min_ | 12 | 121552 | 5.0 | 0.00 | * | 0.00 |
| degree:method:p_min_ | 24 | 121552 | 2.7 | 0.00 | * | 0.00 |
| type:degree:method:p_min_ | 24 | 121552 | 2.9 | 0.00 | * | 0.00 |
